# Biodegradable Nanoprobe for NIR‐II Fluorescence Image‐Guided Surgery and Enhanced Breast Cancer Radiotherapy Efficacy

**DOI:** 10.1002/advs.202104728

**Published:** 2022-02-16

**Authors:** Rui‐Qin Yang, Pei‐Yuan Wang, Kang‐Liang Lou, Yong‐Ying Dang, Hai‐Na Tian, Yang Li, Yi‐Yang Gao, Wen‐He Huang, Yong‐Qu Zhang, Xiao‐Long Liu, Guo‐Jun Zhang

**Affiliations:** ^1^ Cancer Center and Department of Breast and Thyroid Surgery Xiang'an Hospital of Xiamen University School of Medicine Xiamen University Xiamen Fujian 361100 China; ^2^ Key Laboratory for Endocrine‐Related Cancer Precision Medicine of Xiamen Xiang'an Hospital of Xiamen University Xiamen Fujian 361100 China; ^3^ Key Laboratory of Design and Assembly of Functional Nanostructures Fujian Institute of Research on the Structure of Matter Chinese Academy of Sciences Fuzhou Fujian 350000 China; ^4^ The United Innovation of Mengchao Hepatobiliary Technology Key Laboratory of Fujian Province Mengchao Hepatobiliary Hospital of Fujian Medical University Fuzhou Fujian 350025 China; ^5^ Cancer Research Center School of Medicine Xiamen University Xiamen Fujian 361100 China; ^6^ Xiamen Research Center of Clinical Medicine in Breast & Thyroid Cancers Xiamen Fujian 361100 China; ^7^ Department of Biomaterials College of Materials Research Center of Biomedical Engineering of Xiamen and Key Laboratory of Biomedical Engineering of Fujian Province and Fujian Provincial Key Laboratory for Soft Functional Materials Research Xiamen University Xiamen Fujian 361005 China

**Keywords:** biodegradable, breast cancer, image‐guided surgery, NIR‐II fluorescence imaging, radiotherapy sensitization

## Abstract

Positive resection margin frequently exists in breast‐conserving treatment (BCT) of early‐stage breast cancer, and insufficient therapeutic efficacy is common during radiotherapy (RT) in advanced breast cancer patients. Moreover, a multimodal nanotherapy platform is urgently required for precision cancer medicine. Therefore, a biodegradable cyclic RGD pentapeptide/hollow virus‐like gadolinium (Gd)‐based indocyanine green (R&HV‐Gd@ICG) nanoprobe is developed to improve fluorescence image‐guided surgery and breast cancer RT efficacy. R&HV‐Gd exhibits remarkably improved aqueous stability, tumor retention, and target specificity of ICG, and achieves outstanding magnetic resonance/second near‐infrared (NIR‐II) window multimodal imaging in vivo. The nanoprobe‐based NIR‐II fluorescence image guidance facilitates complete tumor resection, improves the overall mouse survival rate, and effectively discriminates between benign and malignant breast tissues in spontaneous breast cancer transgenic mice (area under the curve = 0.978; 95% confidence interval: 0.952, 1.0). Moreover, introducing the nanoprobe to tumors generated more reactive oxygen species under X‐ray irradiation, improved RT sensitivity, and reduced mouse tumor progression. Notably, the nanoprobe is biodegradable in vivo and exhibits accelerated bodily clearance, which is expected to reduce the potential long‐term inorganic nanoparticle toxicity. Overall, the nanoprobe provides a basis for developing precision breast cancer treatment strategies.

## Graphical abstract

A biodegradable diagnostic and therapeutic integrated nanoprobe is constructed for precise breast cancer treatment. The nanoprobe achieves accurate second near‐infrared (NIR‐II) fluorescence image‐guided surgical tumor resection and can be a radiotherapy (RT) sensitizer to enhance breast cancer RT efficacy. In addition, the nanoprobe exhibits in vivo biocompatibility and biodegradation.

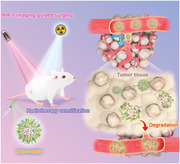

## 1 Introduction

Breast conservation surgery (BCS) followed by whole‐breast irradiation provides excellent tumor control and is recognized as the standard therapeutic option for early‐stage breast cancer.^[^ ^1^ ^]^ Because negative margins optimize patient benefits by minimizing local tumor recurrence and distant metastasis, tumor‐free surgical margins are critical in BCS.^[^ ^2^ ^]^ However, intraoperatively surgically identifying tumor boundaries mainly relies on palpation and visual inspection. Without intraoperative frozen‐section analysis, which greatly increases surgical time and exhibits low sensitivity (65–78%),^[^ ^3^ ^]^ ≈20–40% of patients will undergo further surgical procedures owing to positive margins,^[^ ^4^ ^]^ leading to higher surgical risks and psychological and physical burdens. Meanwhile, intraoperative margin assessment remains a dilemma for conventional imaging modalities such as X‐ray specimen radiography, magnetic resonance imaging (MRI), and computed tomography (CT), which exhibit limited sensitivity and signal specificity and are difficult to apply in the operating room.^[^ ^5^ ^]^ Therefore, a real‐time, high‐resolution, and highly specific method of intraoperatively assessing margins is urgently needed.

Recently, second near‐infrared (NIR‐II) window (1000–1700 nm) fluorescence imaging, which exhibits a high signal‐to‐noise ratio (SNR) and deep tissue penetration, has emerged as a promising strategy for precise image‐guided tumor surgery. To date, NIR‐II contrast agents have included quantum dots,^[^ ^6^ ^]^ single‐walled carbon nanotubes,^[^ ^7^ ^]^ rare‐earth‐doped nanoparticles,^[^ ^8^ ^]^ and organic dyes;^[^ ^9^ ^]^ however, none of them has been approved for clinical application. Moreover, the higher accumulation, long reticuloendothelial system (RES) retention, and inability to bodily excrete such contrast agents has led to dilemmas in developing inorganic NIR‐II nanoprobes.^[^ ^10^ ^]^ Intriguingly, clinically approved indocyanine green (ICG) generated long off‐peak NIR‐II emission spectra with biocompatibility, although the emission peak was not in the NIR‐II window, it still had high quantum yields (QYs) in the off‐peak region, especially higher than the most of NIR‐II inorganic nanoprobes.^[^ ^11^ ^]^ Unfortunately, in current fluorescence image‐guided surgery practice, ICG typically experienced instability and self‐aggregation in aqueous solution, rapid agglomeration and elimination from the body because it nonspecifically binds to proteins.^[^ ^12^ ^]^ Furthermore, due to the lack of tumor targeting of ICG, the sensitivity and specificity for delineating tumor margins are insufficient.^[^ ^13^ ^]^ Therefore, these ICG shortcomings must be overcome to intraoperatively precisely differentiate tumors and healthy tissue in real time.

Furthermore, radiotherapy (RT) is a common palliative treatment way for advanced breast cancer. However, clinical RT success is limited by insufficient damage to tumor deoxyribonucleic acid (DNA)^[^ ^14^ ^]^ mostly because the radiation beam intensity must be curbed to minimize the inevitable collateral damage to healthy tissues, leaving only a trace of ionizing radiation to be absorbed by tumor tissues.^[^ ^15^ ^]^ Thus, one way to overcome these obstacles is to increase RT efficacy by introducing radiosensitizers to tumors. Owing to its high atomic number (*Z* = 64), gadolinium (Gd) exhibits dose‐dependent RT enhancement because of photoelectric and Compton scattering under high‐intensity X‐ray irradiation.^[^ ^16^ ^]^ Therefore, Gd‐based radiosensitizers locally deposited at tumor could enhance X‐ray efficiency, thereby requiring the radiation dose to be decreased and resulting in less collateral damage to the surrounding healthy tissues.^[^ ^17^ ^]^ Accordingly, biosafe Gd‐based nanosensitizers urgently must be developed and used to palliative RT for advanced breast cancer patients.

**Figure 1:**
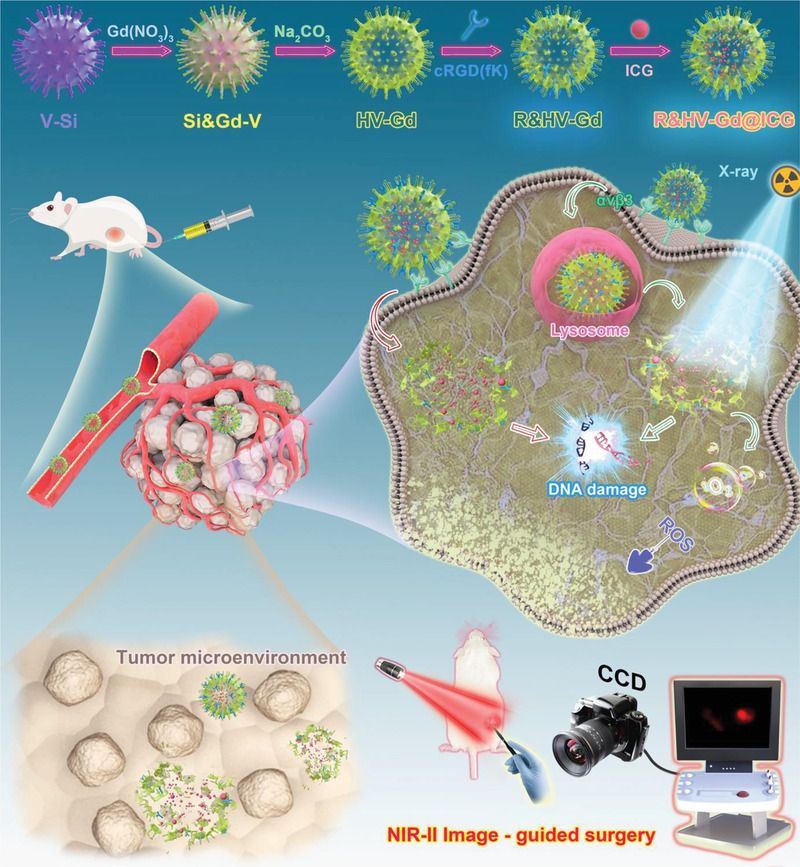
Schematic illustrating R&HV‐Gd@ICG fabrication for NIR‐II image‐guided breast cancer surgery and enhanced RT efficacy.

Therefore, we constructed a Gd‐based degradable hollow virus‐like nanoparticle via the hard template of mesoporous SiO_2_ with loading ICG and modified the nanoparticle surface with cyclic RGD pentapeptide (cRGD(fK)) to develop an R&HV‐Gd@ICG tumor‐targeting nanoprobe (**Figure** **1**). Intriguingly, the R&HV‐Gd nanoparticles remarkably improved both ICG photostability and photobleaching. The nanoprobe accurately illuminates tumors by binding to the integrin *α*_v_ *β*_3_ receptor and exhibiting an SNR of up to 6. Moreover, the in vivo results show that nanoprobe NIR‐II fluorescence imaging can accurately intraoperatively recognize residual tumors, which can be precisely removed to improve the overall survival rate of mouse models. Additionally, the nanoprobe acts as an MRI contrast agent, which outperforms clinically used gadoteric acid meglumine (GAM). Introducing R&HV‐Gd@ICG to tumors effectively enhanced RT efficacy by generating numerous reactive oxygen species (ROSs) to reduce the tumor burden. Notably, the biodegradable nanoparticles gradually formed nanogranules, which promoted tumor permeation and facilitated bodily clearance. In summary, R&HV‐Gd@ICG exhibited biocompatibility, biodegradation, and superior NIR‐II image‐guided tumor surgery and radiation sensitization capabilities with great potential for further clinical applications.

## 2 Results

### 2.1 Preparation and Characterization of R&HV‐Gd@ICG

**Figure 2:**
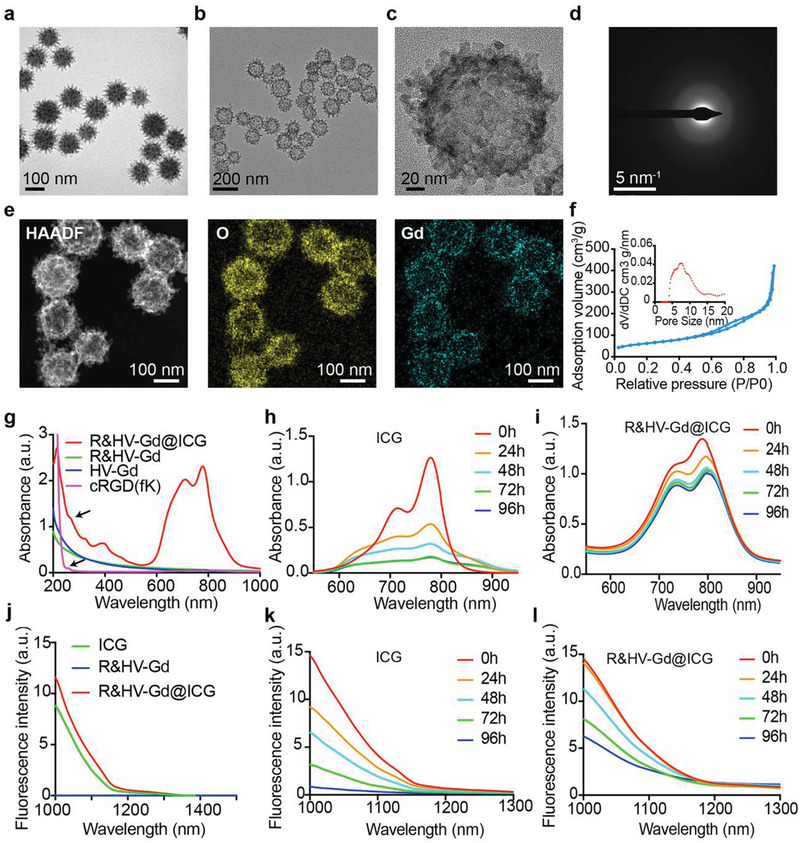
Nanoprobe synthesis and characterization. TEM images of a) Si‐V and b) HV‐Gd. HV‐Gd c) high‐resolution TEM and d) diffraction images. e) HAADF‐STEM image and corresponding HV‐Gd elemental mapping. f) HV‐Gd pore‐size distribution curve and nitrogen adsorption–desorption isotherms. g) UV–NIR absorption spectra for cRGD(fK), HV‐Gd, R&HV‐Gd, and R&HV‐Gd@ICG. Changes in absorption and fluorescence spectra for h,k) free ICG and i,l) R&HV‐Gd@ICG in aqueous solution for 96 h, respectively. j) Fluorescence emission spectra generated in NIR‐II window for ICG, R&HV‐Gd, and R&HV‐Gd@ICG.

Transmission electron microscopy (TEM) images showed that the virus‐like silica (V‐Si) exhibits excellent monodispersity and a spherical structure surrounded by nanospike morphology (**Figure** **2**a). Then, gadolinium nitrate precursors (Gd(NO_3_)_3_·6H_2_O) were reduced by the weak organic base, methenamine, on the V‐Si template surface. Subsequently, the inner silicon template was etched using sodium hydroxide to obtain *⌀* ≈ 140 nm uniform hollow mesoporous Gd nanospheres (HV‐Gd) exhibiting surface‐anchored tubular spines (Figure 2b). Moreover, the scanning electron microscopy (SEM) image also showed that the hollow nanoparticles maintained the rough V‐Si surface (Figure S1, Supporting Information). Furthermore, HV‐Gd was analyzed using high‐resolution TEM (HRTEM). Clearly, the building units consist of interpenetrated nanogranules (Figure 2c), and the absence of lattice fringes indicates that the nanogranules are amorphous, which was further confirmed by the characteristic diffuse halo in the selected‐area electron diffraction pattern (Figure 2d). The elemental mapping images showed that both Gd and O were homogeneously distributed in the hollow nanoparticles (Figure 2e). The isotherm N_2_ adsorption branch showed that HV‐Gd exhibited 7.5 nm mesopores corresponding to the porosity formed when the V‐Si hard template nanospikes were stacked during nanocasting (Figure 2f). The HV‐Gd specific surface area was determined as ≈273.3 m^2^ g^−1^ according to conventional Brunauer–Emmett–Teller calculations, indicating that HV‐Gd could facilitate pharmaceutical drug delivery applications (Figure 2f). We further confirmed the HV‐Gd Gd content by the characteristic binding energies at 1120.00 eV (Gd 3d*3*/*3*) and 1185.00 eV (Gd 3d*5*/*3*) by X‐ray photoelectron spectroscopy (XPS) (Figure S2, Supporting Information), which agreed well with the elemental mapping images. Subsequently, ICG was encapsulated in the hollow Gd‐based nanoshells (HV‐Gd@ICG), which were further amino‐modified using amino silane, and cRGD(fK) was anchored on the HV‐Gd surface by a common 1‐(3‐dimethylaminopropyl)‐3‐ethylcarbodiimide hydrochloride (EDC)/*N*‐hydroxysuccinimide (NHS) reaction (R&HV‐Gd@ICG). The ICG loading efficiency was calculated as 10.44%. Moreover, the R&HV‐Gd@ICG ICG, Gd, and cRGD(fK) molar ratios were determined using the UV–vis–NIR spectrophotometer and inductively coupled plasma (ICP) as 6.1:92.3:1, respectively. The intermediate and final products were water‐soluble and uniformly dispersed (Figure S3, Supporting Information). Particle size was then analyzed using dynamic laser scattering, and the results showed that the V‐Si, HV‐Gd, and R&HV‐Gd@ICG were 143, 145, and 150 nm, respectively, suggesting that ICG loading and peptide modification both negligibly affected the HV‐Gd particle size (Figure S4, Supporting Information). UV–NIR spectral analysis showed that R&HV‐Gd@ICG exhibited an absorption peak characteristic of the cRGD(fK) peptide at 280 nm, suggesting that the cRGD(fK) peptide had been modified on the nanoshell surface (Figure 2g). In addition, R&HV‐Gd@ICG and ICG both exhibited a strong absorption peak at 780 nm, indicating that ICG had been loaded into the cavity (Figure 2g). Furthermore, the results showed that under 808 nm laser excitation with 1000 nm long pass filters, R&HV‐Gd@ICG and ICG both exhibit NIR‐II spectral features (>1000 nm) (Figure 2j). Moreover, the change in the surface potential of nanoprobe was investigated under different synthesis conditions, and the surface potential of R&HV‐Gd@ICG nanoprobe was −18.5 ± 0.65 V (Figure S5, Supporting Information). Because ICG is unstable in aqueous solutions, the absorption and fluorescence spectra were subsequently measured for ICG and R&HV‐Gd@ICG in water. Consequently, free ICG lost ≈70% of its initial absorption and 90% of its initial fluorescence intensities over 96 h (Figure 2h,k). By contrast, R&HV‐Gd@ICG retained more than 70% of its initial absorption and 50% of its initial fluorescence intensities at 96 h (Figure 2i,l). Remarkably, the results suggested that the ICG aqueous stability was notably improved when ICG was encapsulated in R&HV‐Gd. More interestingly, we used an 808 nm laser to continuously irradiate R&HV‐Gd@ICG and ICG, and the results showed that ICG was gradually photobleached only under only laser irradiation for 30 min, while the R&HV‐Gd@ICG fluorescence intensity was fascinatingly maintained even after 30 min of continuous irradiation (Figure S6, Supporting Information). These results suggest that the R&HV‐Gd hollow nanoparticles could remarkably stabilize ICG, which will be very advantageous in long‐term intraoperative imaging.

Because the unique virus‐like metallic oxide nanoshell was constructed using interpenetrating Gd oxide nanogranules, R&HV‐Gd@ICG presented pH‐sensitive decomposition. TEM was used to record the morphology of R&HV‐Gd@ICG incubated in aqueous solutions at pH 7.4, 6.5, and 5.5 for various times. The results showed that the nanoprobe exhibited pH‐dependent structural collapse, slowly degraded in a normal physiological environment (pH 7.4), and remained relatively structurally stable up to 96 h. Meanwhile, the nanoprobe exhibited time‐dependent degradation in the intracellular environment (pH 5.0) and the tumor microenvironment (pH 6.5) and completely degraded in those environments at 12 and 36 h, respectively (Figure S7, Supporting Information). The pH‐sensitive degradation could mainly be attributed to the formation of HV‐Gd nanoparticles in the methenamine solution. That is, Gd_2_O_3_ nanogranules were first generated and then aggregated on the V‐Si surface to form virus‐like stacking bundles (V‐Si@Gd).^[^ ^18^ ^]^ However, because the temperature and pH could not facilitate the subsequent Ostwald ripening required for crystallization,^[^ ^19^ ^]^ an amorphous Gd_2_O_3_ assembly was finally obtained. Interestingly, the stacking force between the nanogranules is often derived from dipole–dipole attractions, which are vulnerable to weakly acidic environments.^[^ ^20^ ^]^ To verify our hypothesis, we analyzed the components degraded in the pH 5.0 buffer for 24 h. The degraded products were divided into nitrated and non‐nitrated groups, and the [Gd^3+^] in sample was detected using ICP. The results showed that after degradation, the free [Gd^3+^] in the non‐nitrated group was very low, accounting for only ≈4% of the total [Gd^3+^] in the nitrated group (Figure S8, Supporting Information), indicating that Gd mainly existed as oxides after degradation. The particle‐size distribution showed that the degradation products contained ≈6.5 nm nanogranules (Figure S9, Supporting Information). Furthermore, massive nanogranules were also detected using HRTEM (Figure S10, Supporting Information), suggesting that the R&HV‐Gd@ICG nanoparticle stacking force could be destructive at a weakly acidic pH. To investigate the pH‐triggered release performance of the ICG from R&HV‐Gd@ICG in different pH (5.0, 6.5, 7.4) environments. As shown in Figure S11 of the Supporting Information, no obvious agglomeration and precipitation occurred after the degradation of nanoparticles, and the amount of ICG released from nanoprobe was below 35% after 24 h incubation at the pH of 7.4. By contrast, a notable release of the loaded ICG from R&HV‐Gd@ICG was achieved when the pH value was decreased to 6.5. 24 h after incubation under the pH value of 5.0, the amount of ICG released reached up to 80%. In addition, we observed the changes in the fluorescence spectra generated for R&HV‐Gd@ICG in different pH buffers. As shown in Figure S12 of the Supporting Information, the absorption and emission spectra negligibly changed for nanoparticles incubated in pH 5.5 and 7.4 solutions, respectively, indicating that the degradation of R&HV‐Gd@ICG had negligibly affected the NIR‐II fluorescence of the released ICG.

### 2.2 R&HV‐Gd@ICG Cellular Uptake and Intracellular ROS Generation

**Figure 3:**
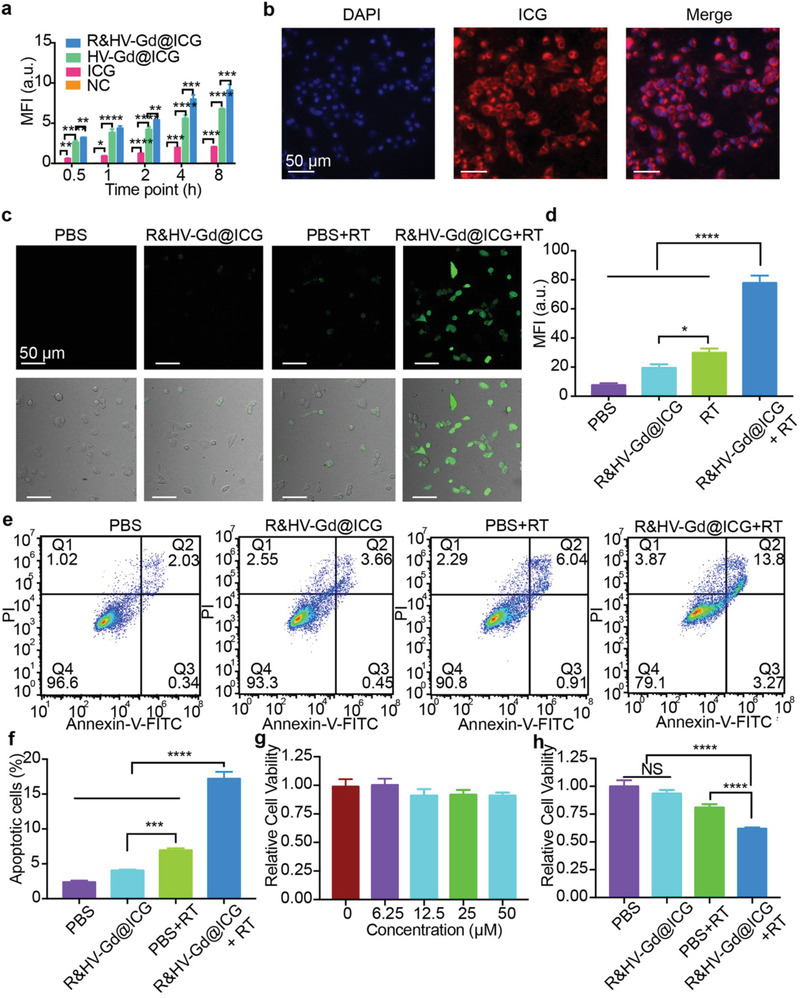
In vitro R&HV‐Gd@ICG cellular uptake and RT sensitization. a) Mean fluorescence intensity obtained by analyzing flow cytometry of 4T1 cells incubated with PBS, ICG, HV‐Gd@ICG, and R&HV‐Gd@ICG at selected time points (*n* = 3). b) Fluorescence scanning micrographs of 4T1 cells incubated with R&HV‐Gd@ICG and DAPI. c) Confocal laser‐scanning microscope images of intracellular ROS obtained using ROS probe H_2_DCFDA in 4T1 cells treated with PBS and R&HV‐Gd@ICG with or without X‐ray irradiation (8 Gy). d) Mean fluorescence intensity quantified based on panel D and using ImageJ software (*n* = 3). e) Flow cytometry analysis and f) quantified apoptosis rates of 4T1 cells treated with PBS and R&HV‐Gd@ICG under X‐ray (8 Gy) irradiation. Nonirradiated PBS and R&HV‐Gd@ICG were set as control group (*n* = 3). g) Viability of 4T1 cells treated with R&HV‐Gd@ICG at various concentrations (*n* = 4). h) Viability of 4T1 cells treated with PBS and R&HV‐Gd@ICG at ([Gd^3+^] =25 × 10^−6^ m) under X‐ray (8 Gy) irradiation. Nonirradiated PBS and R&HV‐Gd@ICG were set as control group (*n* = 4). Data were shown as means ± SD, statistical significance is assessed using one‐way ANOVA followed by Tukey's multiple comparisons test, *p < 0.05, **p < 0.01, ***p < 0.001, ****p < 0.0001. NS, no statistical significance.

The *α*_v_ *β*_3_‐specificity retained by R&HV‐Gd@ICG was confirmed using flow cytometry assays in the 4T1 cells. First, the integrin *α*_v_ *β*_3_ receptor expression in 4T1 cells was estimated using flow cytometry, as shown in Figure S13 of the Supporting Information, which showed higher integrin *α*_v_ *β*_3_ receptor expression. Therefore, the 4T1 cells were treated with R&HV‐Gd@ICG, HV‐Gd@ICG, and free ICG for various times, and the MFI of the cells in the R&HV‐Gd@ICG‐treated group was higher than that of the cells in the other groups at different time points (**Figure** **3**a; Figure S14, Supporting Information). We further studied the 4T1 cell uptake of nanoparticle by fluorescent microscopy, and the results showed that the nanoparticles were efficiently taken up into the cytoplasm (Figure 3b). To investigate the advantageous of virus‐like nanoparticle for cell membrane adhesion, we constructed ICG‐loaded hollow mesoporous silicon nanoparticles (H‐MSNs) as the control, and TEM images showed that the size of H‐MSN was very similar with the size of the HV‐Gd nanoparticles (≈140 nm), while the surface was smooth (Figure S15, Supporting Information). The 4T1 cells were then treated with H‐MSN@ICG and HV‐Gd@ICG. As shown in Figure S15 of the Supporting Information, weak fluorescence signals were observed for HV‐Gd@ICG treatment after 10 min. By contrast, the signals were not detected in H‐MSNs@ICG until 30 min. Moreover, the HV‐Gd@ICG incubated group exhibited very strong ICG fluorescence after incubation for 1 and 2 h comparing with the H‐MSN@ICG group, suggesting that the virus‐like morphology and the RGD peptide both could facilitate cell internalization.

To evaluate the R&HV‐Gd@ICG RT sensitization, we employed 2ʹ,7ʹ‐ dichlorodihydrofluorescein diacetate (H_2_DCFDA) to detect intracellular ROSs. Upon X‐ray irradiation (8 Gy), 4T1 cells incubated with R&HV‐Gd@ICG exhibited significantly more‐intense intracellular fluorescence than the 4T1 cells incubated with PBS (*p* < 0.0001). Meanwhile, the nonirradiated PBS‐ and R&HV‐Gd@ICG‐treated groups exhibited negligible fluorescence signals (Figure 3c,d). These results showed that R&HV‐Gd@ICG had drastically increased intracellular ROS generation upon X‐ray irradiation. Because more than 50% of the DNA damage in standard RT is due to •OH,^[^ ^21^ ^]^ •OH generation was further confirmed using the commercial probe, hydroxyphenyl fluorescein (HPF), which can emit 515 nm green fluorescence after reacting with generated •OH. As shown in Figure S16 of the Supporting Information, the HPF fluorescence in R&HV‐Gd@ICG solution was significantly more intense than the deionized (DI) water under the same X‐ray irradiation (*p* < 0.0001). Furthermore, upon X‐ray irradiation, 4T1 cells incubated with R&HV‐Gd@ICG showed stronger intracellular HPF fluorescence than those incubated with PBS (*p* < 0.0001). Meanwhile, the nonirradiated PBS‐ and R&HV‐Gd@ICG‐treated groups both exhibited negligible fluorescence (Figure S17, Supporting Information), demonstrating that R&HV‐Gd@ICG had generated a massive concentration of •OH radicals under X‐ray irradiation. These results inspired us to evaluate post‐RT cell apoptosis. The results showed that R&HV‐Gd@ICG‐treated 4T1 tumor cells induced remarkably higher cell apoptosis after X‐ray irradiation compared to the other groups (Figure 3e,f). In addition, we detected the cytotoxicity of various RGD&HV‐ICG concentrations toward 4T1 cells. Concentrations up to 50 × 10^−6^ m [Gd^3+^] exhibited no obvious cytotoxicity toward tumor cells, indicating the excellent nanoparticle biocompatibility (Figure 3g). However, upon X‐ray irradiation, the nanoparticles were significantly more cytotoxic than the radiation alone, indicating that the nanoparticle‐mediated RT could generate a massive concentration of cytotoxic hydroxyl radicals to kill tumor cells (*p* < 0.0001) (Figure 3h). Moreover, we performed a cell cloning assay to further evaluate the long‐term R&HV‐Gd@ICG radiation sensitization, as shown in Figure S18 of the Supporting Information. The X‐ray‐irradiated 4T1 tumor cells incubated with nanoparticles exhibited only a few viable cell colonies, which are significantly fewer than the radiation‐only group (*p* < 0.001). By contrast, the nonirradiated PBS and nanoparticle treated groups both exhibited abundant cell colonies. These results showed that R&HV‐Gd@ICG could meaningfully sensitize radiation to inhibit tumor cell proliferation, making R&HV‐Gd@ICG as a potential RT sensitizer for clinically eliminating breast cancer.

### 2.3 In Vitro and In Vivo R&HV‐Gd@ICG MRI

**Figure 4:**
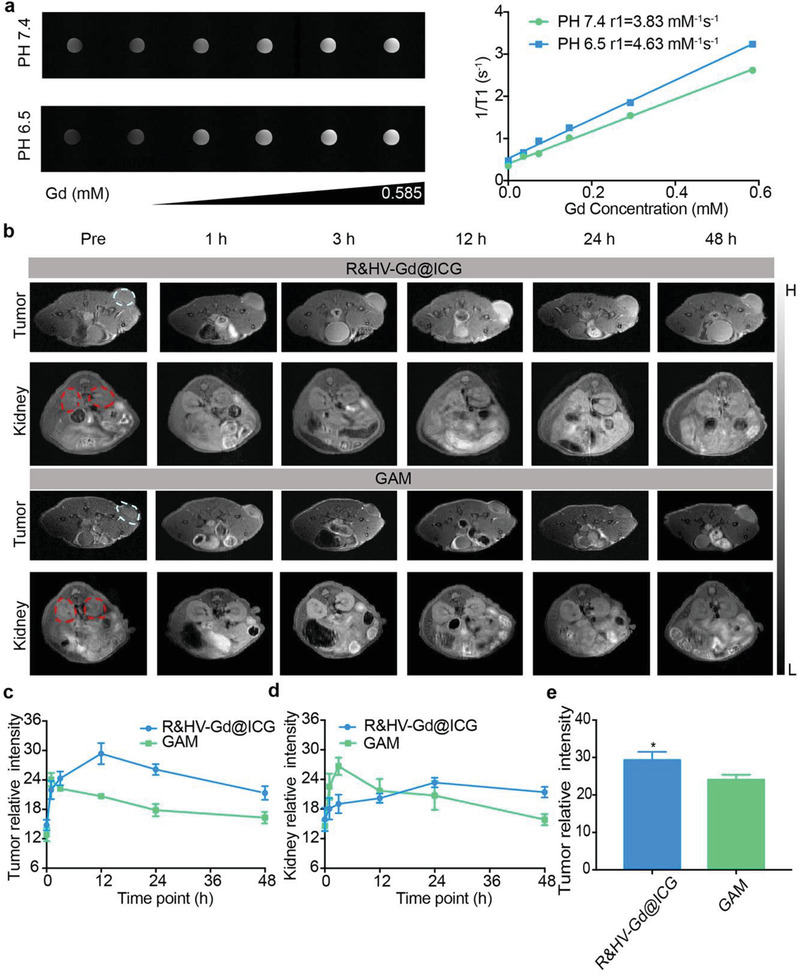
In vitro and in vivo R&HV‐Gd@ICG MRIs. a) T1‐weighted MRIs (left) and longitudinal relaxivities (r_1_) (right) for R&HV‐Gd@ICG recorded using 1.5‐T MRI scanner at pH 6.5 and 7.4. b) MRIs of mice intravenously injected with R&HV‐Gd@ICG or Gadoteric Acid Meglumine (GAM) ([Gd^3+^] = 25 × 10^−6^ m kg^−1^) at different time points. White and red circles indicate tumor and kidney, respectively (*n* = 3). Relative c) tumor and d) kidney background signal intensities based on panel MRIs generated at selected time points (*n* = 3). e) Comparison of relative maximum tumor background signal intensities obtained for mice injected with R&HV‐Gd@ICG and GAM (*n* = 3, data were shown as means ± SD, statistical significance is determined by two‐tailed unpaired *t*‐test, *p < 0.05).

Because of its five unpaired 3d electrons, Gd can be used as an MRI T1‐shortening agent. Thus, we compared the clinical GAM and R&HV‐Gd@ICG MRI performances in vitro. The results showed that under a 1.5‐T magnetic field, T1‐MRIs exhibited concentration‐dependent R&HV‐Gd@ICG brightening at both pH 7.4 and 6.0, with longitudinal relaxivity (r_1_) values of 3.83 and 4.63, respectively (**Figure** **4**a). Interestingly, the R&HV‐Gd@ICG r_1_ value was higher than the GAM one (3.83 vs 3.03, respectively) under normal pH conditions, indicating that the Gd oxide composition negligibly influenced the Gd^3+^ MRI contrastability (Figure S19, Supporting Information). Even though, owing to the amorphous state of HV‐Gd aggregation, the surface package of APETES and cRGD(fK) peptides that reduced the interface between Gd ions and water, the r1 value of our nanoprobes was noncomparable with those of other Gd‐based nanocrystals (r1 = 5.53),^[^ ^22^ ^]^ but our HV‐Gd nanoparticles have comparable r1 relaxivity (3.83) with Gd‐based small molecule contrast agent (3.03) for future potential clinical application as MRI contrast agents. Subsequently, 4T1‐tumor‐bearing mice were intravenously injected with R&HV‐Gd@ICG or GAM at the same Gd^3+^ doses. As shown in Figure 4b, the tumor region MRI signal was considerably more intense relative to that of the control in both groups. The quantitative analysis revealed that the tumor MRI signal of the R&HV‐Gd@ICG‐treated group reached the maximum at 12 h postinjection and was maintained up to 48 h. Meanwhile, the tumor signal of the GAM‐treated group reached the maximum at 1 h postinjection and decayed very quickly (Figure 4c). Additionally, GAM was rapidly eliminated mainly in the kidneys, while (more importantly) the MRI signal in the kidney of R&HV‐Gd@ICG group gradually intensified, indicating that HV‐Gd may be partially excreted through the kidneys (Figure 4d). Furthermore, the most intense tumor signal in the R&HV‐Gd@ICG‐treated group was even much stronger than that in the GAM‐treated one (Figure 4e). Therefore, the in vitro and in vivo results both suggested that R&HV‐Gd@ICG exhibited better MRI performance than GAM.

### 2.4 In Vivo and Ex Vivo NIR‐II Fluorescence Imaging and Biodistribution

**Figure 5:**
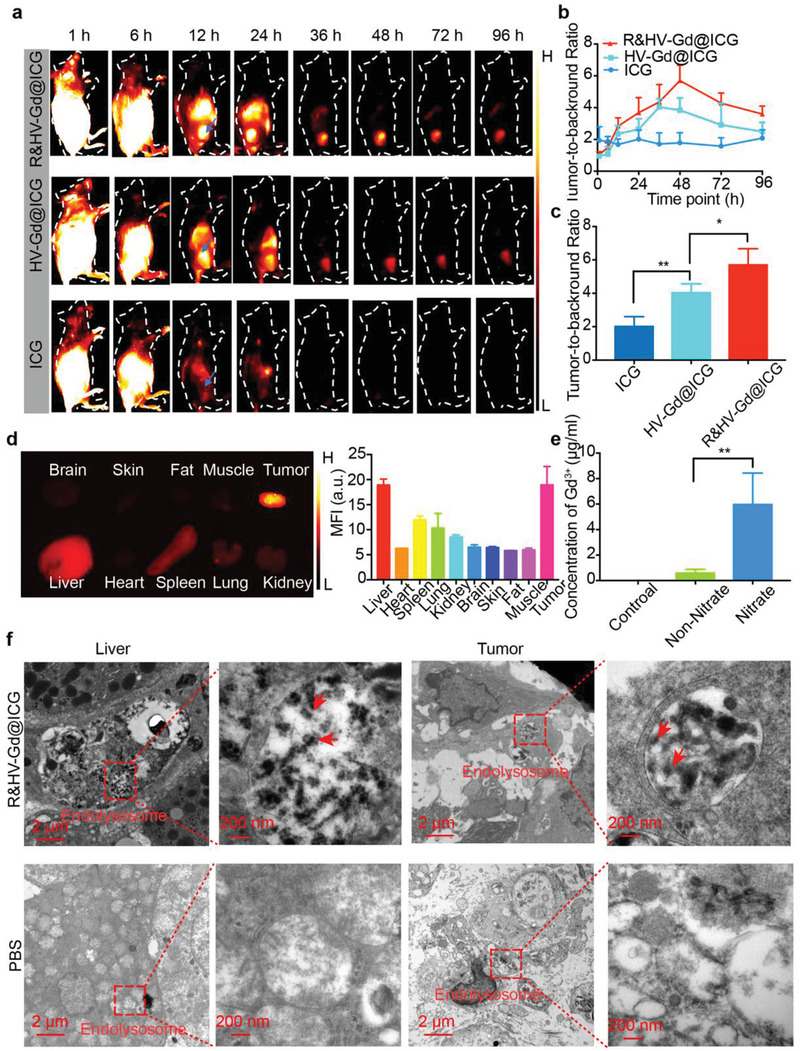
Validation of target specificity and R&HV‐Gd@ICG biodistribution in 4T1‐tumor‐bearing mouse models. a) NIR‐II fluorescence images of mice intravenously injected with R&HV‐Gd@ICG, HV‐Gd@ICG, and ICG and b) TBR concentration plotted as function of time for 4T1‐tumor‐bearing mice (*n* = 4). c) Comparison of individual R&HV‐Gd@ICG, HV‐Gd@ICG, and free‐ICG dye specific tumor‐targeting capabilities at maximum TBR (*n* = 4). d) NIR‐II fluorescence images (left) of organs and tumors excised 48 h after mice were injected with R&HV‐Gd@ICG and corresponding semiquantitative analysis of mean sample fluorescence intensity (right) (*n* = 3). e) Gd^3+^ concentrations in non‐nitrified and nitrified urine samples collected from mice injected with R&HV‐Gd@ICG. PBS‐injected mice were set as control group (*n* = 3). f) TEM images of liver and tumor 12 h after mice were intravenously injected with R&HV‐Gd@ICG or PBS. Red arrows indicate degraded nanoparticles. Data were shown as means ± SD, statistical significance is assessed using one‐way ANOVA followed by Tukey's multiple comparisons test, *p < 0.05, **p < 0.01.

To further verify the R&HV‐Gd@ICG tumor‐targeting specificity in vivo, 4T1‐tumor‐bearing mice were intravenously injected with R&HV‐Gd@ICG, HV‐Gd@ICG, and free ICG. NIR‐II fluorescence imaging showed that in the free‐ICG group, the fluorescence signal intensified primarily in the liver, whereas only a weak signal was observed in the tumor over time. By contrast, the R&HV‐Gd@ICG group showed a strong fluorescence signal in the tumor, and the signal intensity was retained up to 96 h postinjection (**Figure** **5**a). Furthermore, the maximum SNR in the R&HV‐Gd@ICG group at 48 h postinjection (5.7 ± 1.0) was higher than those in the HV‐Gd@ICG group at 36 h postinjection (4.0 ± 0.5) and in the ICG group (2.0 ± 0.6) at 24 h postinjection, indicating both the R&HV‐Gd@ICG *α*_v_ *β*_3_‐specific targeting and the pH‐sensitive ICG release for tumor diffusion and penetration (Figure 5b,c). To further observe the nanoprobe biodistribution in vivo, mouse organs were dissected for fluorescence imaging at 48 h postinjection, as shown in Figure 5d. Clearly, the tumor showed the most intense mean fluorescence signals, whereas the RES organs, liver, lung, and kidney presented medium fluorescence signals because those organs had metabolized and excreted the nanoprobe, and other organs and tissues (i.e., brain, muscle, fat, and skin) exhibited negligible fluorescence signals. To determine the R&HV‐Gd@ICG pharmacokinetic and biodistribution profiles, the blood concentration–time curve showed that the nanoprobe half‐life was 92.06 min (Figure S20, Supporting Information), and the free‐ICG half‐life had greatly improved in the blood. Furthermore, to analyze how the nanoprobe was metabolized in vivo, urine samples were collected from the R&HV‐Gd@ICG‐treated mice 24–36 h postinjection, and the samples were then randomly divided into non‐nitrified and nitrified urine. According to the inductively coupled plasma mass spectrometry (ICP‐MS) analysis results, although negligible Gd^3+^ was detected in nondigested urine, Gd^3+^ was readily detected in the urine digested using nitrohydrochloric acid (Figure 5e), suggesting that the nanoprobe is excreted mainly as Gd_2_O_3_ nanogranules in urine. Moreover, the time‐dependent degradation of the nanoparticles was observed in the intracellular environment (pH 5.0), and the MR imaging and fluorescence imaging showed that the strongest tumor signal at 12 hours postinjection. Therefore, we chose this time point to observe the nanoparticles in the liver and tumor by TEM. TEM images showed that the nanoparticle structure had collapsed and degraded in the lysosome environment in both the liver and tumor tissues (Figure 5f), further demonstrating the nanoprobe biodegradability in vivo. Finally, to more accurately analyze the R&HV‐Gd@ICG biodistribution in the mice, mouse organs were collected at different time points after R&HV‐Gd@ICG had been intravenously administered, and the organs were further evaluated using ICP‐MS. Nearly all the R&HV‐Gd@ICG nanoparticles had been bodily eliminated, with very few remaining in the spleen, liver, and lung tissue after 28 d postinjection with a single dose (Figure S21, Supporting Information), further demonstrating the nanoprobe biosafety.

### 2.5 NIR‐II Fluorescence Image‐Guided Surgery in 4T1‐Tumor‐Bearing Mice

**Figure 6:**
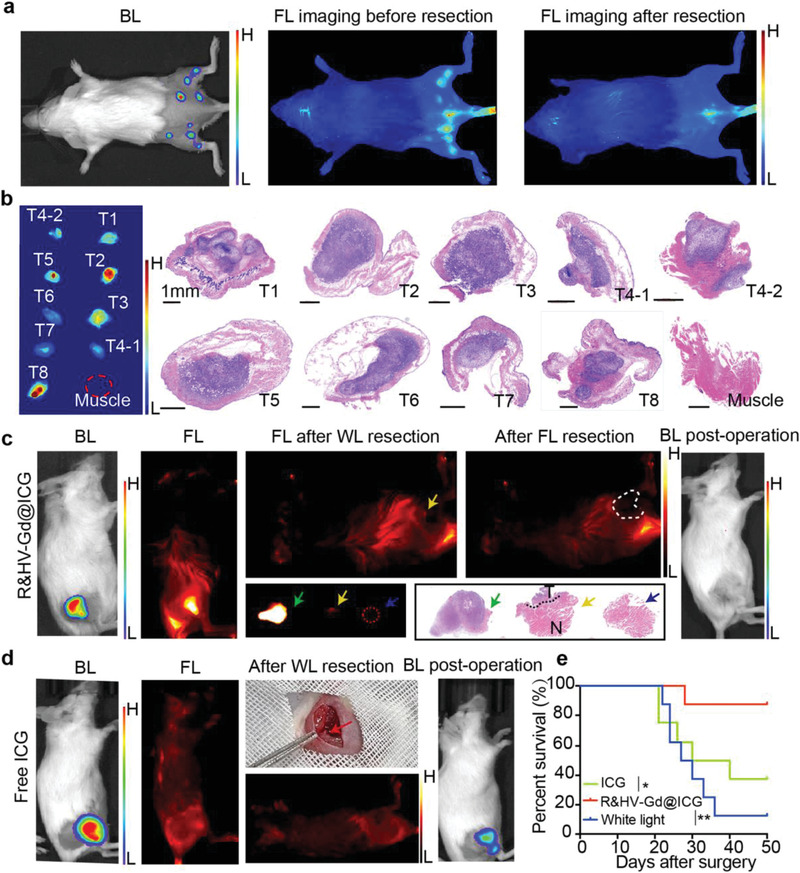
NIR‐II fluorescent image‐guided surgically resected 4T1‐Luc tumors. a) Preoperative bioluminescent, intraoperative, and postoperative fluorescence images taken during tumor resection surgery on representative multiple microtumor mouse model injected with R&HV‐Gd@ICG. b) In vitro fluorescence and corresponding H&E‐stained histological images of tumor tissue. Preoperative, intraoperative, and postoperative images taken during white‐light‐only and NIR‐II fluorescence‐guided tumor resection surgeries on representative mouse models injected with c) R&HV‐Gd@ICG and d) ICG. H&E‐stained histological images of surgical margins showing primarily tumor (green asterisk), residual tumor tissue (yellow asterisk), and negative surgical margin after secondary fluorescence‐guided surgery (black asterisk). e) Kaplan–Meier analysis showing significant difference in survival of R&HV‐Gd@ICG, ICG, and white‐light‐only‐guided surgery groups (*n* = 8, statistical significance was assessed using Log‐rank test, *p < 0.05, **p < 0.01).

Because the highest tumor‐to‐background ratio (TBR) time point was confirmed at 48 h postinjection, NIR‐II fluorescence image‐guided tumor surgery was performed at this time point. First, we constructed a multiple microtumor model to determine whether the fluorescent nanoprobe could intraoperatively recognize small tumors. The results showed that the intraoperative tumor fluorescence signal was highly consistent with the bioluminescence imaging signal (**Figure** **6**a), and the corresponding tissues resected using NIR‐II fluorescence guidance confirmed based on pathology that the entire borderline between the tumor and the healthy tissue could be distinguished (Figure 6b). Among the 31 microtumors confirmed using bioluminescence, 29 were intraoperatively identified using NIR‐II fluorescence imaging (Figure 6a; Figure S22, Supporting Information), indicating that R&HV‐Gd@ICG‐based fluorescence imaging could effectively intraoperatively identify small residual tumors.

Furthermore, to simulate intraoperative tumor identification, we constructed a residual tumor model to evaluate the feasibility of using fluorescence image‐guided surgery to precisely identify residual tumors. As shown in Figure 6c, the residual tumor was accurately identified based on the fluorescence signal after the tumor had been injected with R&HV‐Gd@ICG. NIR‐II fluorescence‐based residual tumor tissue recognition by was confirmed using hematoxylin and eosin (H&E) pathology staining. By contrast, some residual tumors in ICG‐group exhibited no fluorescence signal, demonstrating the fundamental tumor targeting and retention defect of ICG, which is clinically used for tumor surgery (Figure 6d). Finally, after 15 d, the residual tumor was locally evaluated using bioluminescence imaging because the present subcutaneous tumor models constructed by luciferase‐gene‐labeled 4T1 cells. In the R&HV‐Gd@ICG group, only one mouse in eight (1/8, 12.5%) postoperatively exhibited a residual tumor bioluminescence signal. However, four mice in eight (4/8, 50%) postoperatively exhibited a residual tumor bioluminescence signal in the ICG group, indicating that the R&HV‐Gd@ICG exhibited superior performance as NIR‐II fluorescence contrast agents (Figure S23, Supporting Information). In the control group—wherein tumor resection had been guided using only white light, all the mice (8/8, 100%) exhibited tumor recurrence, further suggesting that the residual tumor model had been properly constructed (Figure S23, Supporting Information). Additionally, the Kaplan–Meier survival curve indicated that R&HV‐Gd@ICG‐based fluorescence guidance facilitated the most complete tumor resection and improved the overall mouse survival rate compared to the other groups (Figure 6e).

### 2.6 NIR‐II Fluorescence Imaging in Spontaneous Breast Cancer Mouse Model

**Figure 7:**
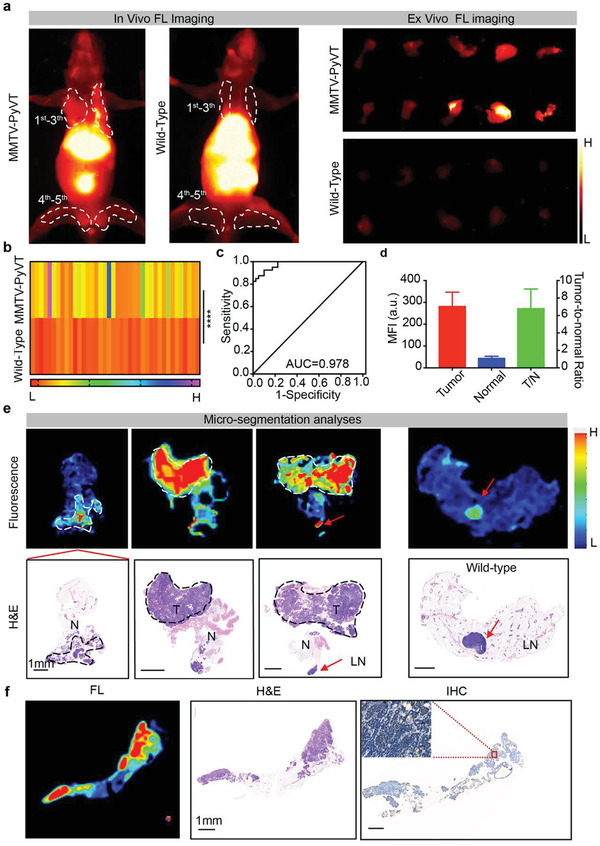
NIR‐II fluorescence imaging in spontaneous breast cancer mouse model. a) In vivo and ex vivo fluorescence images of mammary glands 48 h after MMTV‐PyVT transgenic and wild‐type mice were intravenously injected with R&HV‐Gd@ICG. b) Mean fluorescence intensities for 1st–5th layers of resected mammary gland tissue in MMTV‐PyVT transgenic and wild‐type mice (*n* = 40, statistical significance is determined by two‐tailed unpaired *t*‐test, ****p < 0.0001). c) Fluorescence imaging receiver operating characteristic (ROC) curve to differentiate between normal tissues and cancer tumors. d) Mean tumor TNR plotted as function of mean TNR in healthy breast tissue for transgenic mice, as measured using NIR‐II fluorescence intensity scanning for 10 µm thick tissue slices with Odyssey Imaging System. (Data were shown as means ± SD, *n* = 4.) e) Microscopic biodistribution of R&HV‐Gd@ICG in breast tissues. Upper row shows fluorescence images of 10 µm slices of mouse breast tissue. Lower row shows corresponding H&E staining. Dotted line indicates tumor area; red asterisk, lymph node. f) Representative example of breast tissue showing corresponding fluorescence image (left), H&E section (middle), and immunohistochemical stain of *α*_v_ expression (right).

Finally, R&HV‐Gd@ICG was investigated as an NIR‐II fluorescence contrast agent for application to spontaneous breast tumor surgery navigation. In vivo and ex vivo fluorescence imaging of MMTV‐PyVT mice intravenously injected with R&HV‐Gd@ICG both displayed highly intense mammary gland fluorescence signals, whereas the fluorescence signal in the healthy breast tissue of wild‐type mouse was barely visible (**Figure** **7**a). Subsequently, ex vivo semiquantitative analysis revealed that the breast tumor tissue exhibited a more‐intense fluorescence signal than the healthy breast tissue (Figure 7b), and the area under the curve (AUC) for fluorescence‐based differentiation between malignant and benign tissues was calculated as 0.978 (95% confidence interval [CI]: 0.952, 1.0), indicating that R&HV‐Gd@ICG‐based NIR‐II fluorescence imaging could accurately and intraoperatively distinguish the tumor from the healthy tissue (Figure 7c). To further analyze the nanoprobe distribution in microscopic mammary tissue, tissue sections were scanned using NIR I fluorescence imaging system. Semiquantitative analysis revealed that the fluorescence signal in microscopic tumor area was seven times more intense than the signal in microscopic healthy tissue area (Figure 7d). The tumor section samples were then stained with H&E and analyzed using NIR‐II fluorescence imaging. As shown in Figure 7e, the microscopic R&HV‐Gd@ICG fluorescence images distinctly differentiated between invasive carcinoma and a nearby healthy duct, and it is consistent with the H&E‐staining results and clearly indicates that the nanoprobe can accurately identify tumor margins. Interestingly, lymph nodes in both MMTV‐PyVT mice and wild‐type mammary glands exhibited a highly intense fluorescence signal, indicating that R&HV‐Gd@ICG could be used to guide sentinel lymph node biopsies (Figure 7e). Furthermore, we *α*_v_‐immunostained breast tissue, and the results showed that the fluorescence signal, histological diagnosis, and integrin expression were all strongly correlated, further demonstrating the R&HV‐Gd@ICG reliability for application in NIR‐II fluorescence image‐guided tumor resection (Figure 7f).

### 2.7 In Vivo RT Sensitization

**Figure 8:**
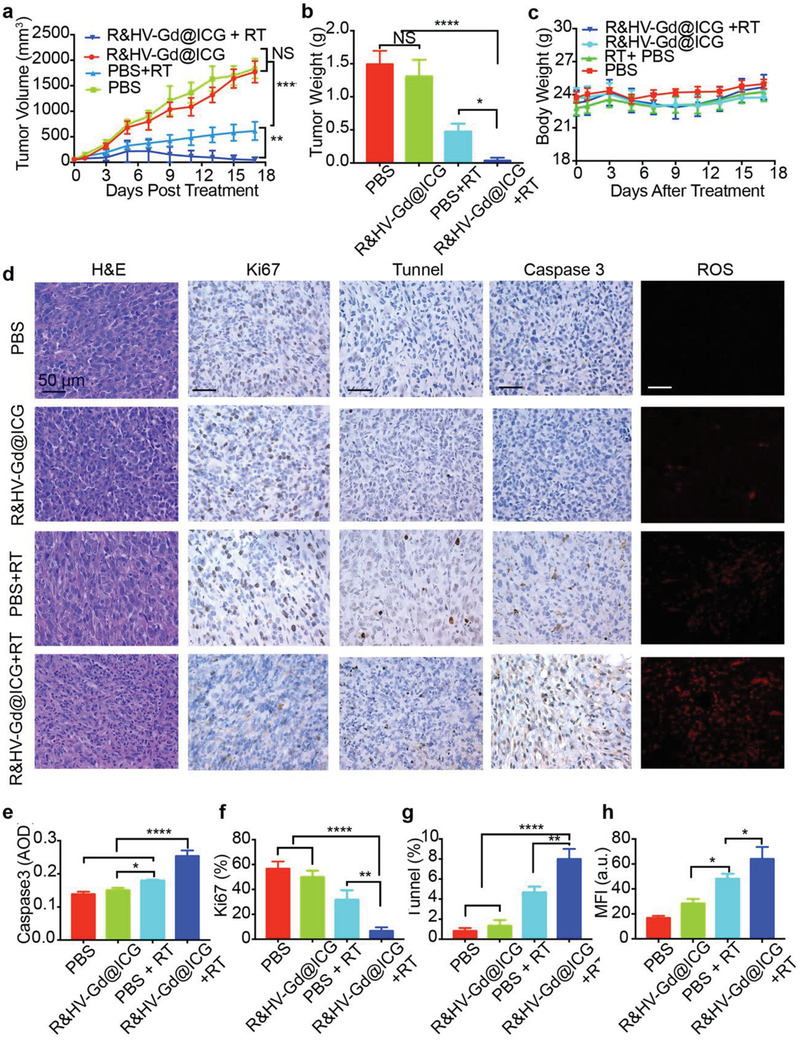
Radiosensitization of R&HV‐Gd@ICG in 4T1‐tumor‐bearing mice. a) Tumor growth curves for mice treated with R&HV‐Gd@ICG and PBS with or without X‐ray RT (*n* = 4). Treatments were conducted on days 0 and 6, and each exposure was 6 Gy. b) Tumor weight after different treatments at day 17 (*n* = 4). c) Bodyweight curves for mice treated with R&HV‐Gd@ICG and PBS with or without irradiation (*n* = 4). d) H&E staining, Ki67, TUNEL, and Caspase3 IHC staining images of tumor slices and ROS immunofluorescence images of tumor slices after various treatments. Corresponding quantitative analyses of e) Caspase 3, f) Ki‐67, g) TUNEL, and h) ROS fluorescent staining after different treatments (*n* = 3). Data were shown as means ± SD, statistical significance is assessed using one‐way ANOVA followed by Tukey's multiple comparisons test, *p < 0.05, **p < 0.01, ****p < 0.0001.

Owing to Gd photoelectric effects, Gd‐based nanoagents can notably increase hydroxyl radical production under X‐ray irradiation. Therefore, tumor elimination efficiency was evaluated for mice that had been intravenously injected with R&HV‐Gd@ICG. As shown in **Figure** **8**a and Figure S24 (Supporting Information), the nonirradiated R&HV‐Gd@ICG‐treated mice exhibited almost no tumor growth inhibition. However, upon X‐ray irradiation, R&HV‐Gd@ICG‐treated mice exhibited efficient RT sensitization and remarkably tumor regression compared to the mice that had only received X‐ray RT. In addition, the excised tumor weights measured on day 17 confirmed that the RT‐sensitized groups exhibited remarkable tumor eradication and inhibited tumor growth compared with the other groups (Figure 8b). Moreover, no difference in bodyweight was noted among the various groups, indicating that R&HV‐Gd@ICG‐based RT sensitization did not cause systemic toxicity in mice (Figure 8c).

Furthermore, we combined immunofluorescence imaging and immunohistochemical (IHC) staining to analyze the antitumor effectiveness of different tumor tissue. As shown in Figure 8d, the H&E‐stained R@HV‐Gd/ICG+RT‐treated tumor sections exhibited the largest tumor necrosis regions, thereby confirming the RT sensitization efficacy. Moreover, the Ki67‐IHC‐stained tumor sections indicated that R&HV‐Gd@ICG+RT‐treated mice exhibited less highly proliferative tumor cells compared with the other groups (Figure 8d,g). TUNEL staining revealed more apoptotic tumor cells in the R&HV‐Gd@ICG + RT treatment group than in the other groups (Figure 8d,h). In addition, compared to all the other groups, the Caspase‐3 activity was drastically enhanced when the R&HV‐Gd@ICG was exposed to X‐ray irradiation (Figure 8d,i). These results showed that the R&HV‐Gd@ICG+ RT treatment was energetically evolved in activating the Caspase‐3 effector, which may restrain cancer cell proliferation and induce cell apoptosis. Finally, the tumor tissue ROS immunofluorescence showed that the nonirradiated R&HV‐Gd@ICG and PBS groups both exhibited almost no tumor fluorescence signal, whereas the mice injected with R&HV‐Gd@ICG and subsequently irradiated with X‐rays exhibited a stronger fluorescence signal than the mice that only received X‐ray RT (Figure 8d,j), suggesting that exposing R&HV‐Gd@ICG to X‐ray irradiation increased the generation of ROS radicals to eradicate tumors.

### 2.8 R&HV‐Gd@ICG Biosafety

One day after the mice were injected with the nanoprobe, the white blood cell and lymphocyte counts slightly increased and returned to normal after 3 d, while the red blood cell and platelet counts showed no obvious change. Meanwhile, one day after the mice were injected with R&HV‐Gd@ICG, the alanine amino transferase, aspartate amino transferase, alkaline phosphatase, creatine, and blood urea nitrogen levels also slightly increased and returned to normal after 3 or 7 d (Figure S25a, Supporting Information). Additionally, the histopathology of the H&E‐stained major organs (i.e., heart, liver, spleen, lung, kidney, and brain) showed no obvious changes in pathology (Figure S25b, Supporting Information). To further evaluate the Gd‐based‐nanoparticle‐induced immune responses in the mice, serum levels of TNF‐*α* and IL‐6 that are inflammation biomarkers were detected using an enzyme‐linked immunosorbent assay (ELISA). As shown in Figure S26 of the Supporting Information, compared with the PBS group, no meaningful changes of serum TNF‐*α* and IL‐6 levels were observed at both 3 and 7 d postinjection. These results demonstrated that R&HV‐Gd@ICG exhibited negligible toxicity in the mice and could, therefore, be clinically applied.

## 3 Discussion

Surgical paradigms shift when surgeons are empowered to perform surgeries faster, more skillfully, and less expensive than current standards.^[^ ^23^ ^]^ In vivo NIR‐II fluorescence imaging is an emerging bioimaging modality that combines high image resolution and tissue penetration to effectively assist surgeons in accurately removing tumors during BCS.^[^ ^24^ ^]^ Interestingly, the United States Food and Drug Administration‐approved ICG can be applied to NIR‐II imaging because ICG exhibits a QY much higher than most synthetic NIR‐II‐emitting contrast agents.^[^ ^25^ ^]^ Therefore, ICG can facilitate the clinical application of in vivo NIR‐II fluorescence imaging. In the present study, R&HV‐Gd remarkably enhanced both ICG photostability and photobleaching because ICG was encapsulated in the nanoparticle cavity and mesoporous channel, indicating that ICG is more conducive to implementing NIR‐II fluorescence image‐guided surgery. Additionally, the cRGD‐peptide‐modified probe can preferentially bind to integrin *α*_v_ *β*_3_, which is a surface membrane protein involved in tumor angiogenesis and is frequently overexpressed in breast cancer tumors compared with healthy tissues.^[^ ^26^ ^]^ Therefore, R&HV‐Gd has endowed ICG with precise tumor target ability. Moreover, an ≈140 nm nanoprobe can effectively prolong ICG blood circulation. Our results showed that the R&HV‐Gd@ICG half‐life was 92.06 min in blood, which was considerably longer than that of free ICG (only ≈3 min), indicating that the tumor nanoprobe accumulation had been effectively improved. Notably, nanoparticles are somewhat contradictory because large nanoparticles are inherently unfavorable for tumor penetration, while small ones exhibit poor tumor retention.^[^ ^27^ ^]^ Many biodegradable drug delivery systems have demonstrated enhanced tumor accumulation and accelerated ICG release, thereby facilitating tumor penetration of ICG.^[^ ^28^ ^]^ In the view of above investigation, tumor‐microenvironment‐induced R&HV‐Gd@ICG degradation could facilitate ICG diffusion and penetration in tumors. Because of the present ingenious ICG delivery strategy, R&HV‐Gd@ICG‐based NIR‐II fluorescence imaging precisely identified small residual tumor lesions and promoted complete tumor resection.

In addition, the nanoparticle metal oxide shell achieved self‐functionalization. Owing to Gd, R&HV‐Gd@ICG acts as an MRI contrast agent, which outperforms commercial MRI contrast agents such as GAM mainly because tumor cell penetration of GAM is difficult and GAM quickly enters and exits tumor blood vessels. By contrast, the virus‐like R&HV‐Gd@ICG surface roughness promoted cell internalization efficiency and dramatically concentrated contrast agents at the tumor^[^ ^29^ ^]^ by combining active and passive delivery mechanisms. Moreover, pH‐sensitive R&HV‐Gd@ICG decomposition facilitated tumor diffusion and penetration for MRI. In addition, combining nanotechnology with targeted delivery strategies has expanded the scope of radiosensitizer development. Notably, owing to the high Gd atomic number, Gd‐based nanomaterials enhanced RT efficacy by boosting ROS production, increasing oxidative stress, and chemically binding DNA.^[^ ^16^, ^30^ ^]^ Additionally, facile tumor‐targeted molecules/protein functionalization on nanomaterial surfaces can selectively accumulate in tumors to precisely target radiosensitization while exempting the surrounding healthy tissues. By contrast, traditional radiation sensitizers such as 5‐Fu lack tumor‐targeting specificity and do not accumulate in tumors, thereby amplifying systemic toxicity.^[^ ^15^, ^31^ ^]^ Therefore, the R&HV‐Gd@ICG nanoprobe has a great potential for application in palliative radiotherapy for advanced breast cancer patients, such as inoperable locally advanced breast cancer, lung metastasis, where sensitization strategies cloud improve the efficacy of radiotherapy.

For inorganic nanoprobes, long‐term cytotoxicity is still a major concern owing to severe RES accumulation, which limits their further clinical applications.^[^ ^32^ ^]^ Although nanoparticles (<5 nm) below the renal clearance threshold can be quickly bodily excreted, short tumor retention times limit nanoparticle application to surgical navigation and RT sensitization. One way to solve these problems is to design biodegradable nanoprobes exhibiting transformable particle sizes. In the present study, the nanoparticles structurally collapsed depending on the environmental pH, and in vivo experiments showed that Gd was detectable in the urine of the mice injected with the nanoprobe and that the Gd^3+^ concentration was dramatically increased in the digested urine, suggesting that the nanoparticles may mostly degrade into Gd_2_O_3_ particles that are excreted through the kidneys. From the biosafety perspective, R@HV‐Gd@ICG‐decomposition‐derived Gd_2_O_3_ nanogranules, unlike Gd^3+^, did not induce toxicity in the mouse models.^[^ ^33^ ^]^ The gadolinium oxide nanoshells could have been heaped up by the nanogranules, and the stacking force could be destroyed in an acidic environment, thereby collapsing the hollow structures. Unfortunately, 28 d after the mice had been injected with the nanoprobe, a low Gd concentration was detected in the major organs mainly because R&HV‐Gd@ICG slowly degrades (>6 d) in a normal physiological environment, which delays nanoprobe excretion. Moreover, the partial degraded nanoparticles may be larger than the renal excretion threshold, resulting in a slower metabolic pathway including urine and fecal excretion. Nevertheless, the present nanoparticles were excreted much faster than previously reported nonbiodegradable large inorganic nanoparticles (90–115 d).^[^ ^34^ ^]^ In addition, we performed a pilot toxicology study to show that the nanoprobe was well tolerated. Although the mouse blood biochemistry panels changed immediately after the mice were injected with the nanoprobe, all the levels returned to within the normal range within one week, and the H&E‐stained major organs showed no obvious changes in pathology. All the results indicated that the nanoprobe showed good biosafety.

In summary, the study results present the potential benefits of a biodegradable, diagnostic, and therapeutic integrated nanoplatform for the precise breast cancer treatment strategies. Combining multimodal imaging with tumor‐targeting strategies can shift the paradigm of surgical oncologic and diagnostic imaging and offer a unique opportunity to improve RT benefits. It also provides a new strategy for the biotransformation application of inorganic nanoparticles.

## 4 Experimental Section

### Preparation of HV‐Gd

First, cetyltrimethylammonium bromide (750 mg, Sigma) was dissolved in DI water (60 mL). Then, sodium hydroxide (0.80 mL, 0.1 m) was added, and the mixture was stirred at 60 °C for 30 min. Subsequently, cyclohexane (16 mL) and tetraethyl orthosilicate (TEOS, 4 mL, Sigma) were added, and the mixture was allowed to react at 60 °C for 72 h. Then, the upper oil phase was removed and washed three times with DI to obtain V‐Si nanoparticles. Then, V‐Si (100 mg) and Gd(NO_3_)_3_·6H_2_O (213.2 mg) were dissolved in DI water (50 mL) and were stirred for 30 min. Subsequently, hexamethylenetetramine (0.105 g, Sigma) was added, and the mixture was stirred at 90 °C for 4 h. Finally, the samples were washed with DI water to obtain virus‐like gadolinium nanoparticles. The virus‐like mesoporous silica template was etched using 0.5 m Na_2_CO_3_, was stirred at 75 °C for 12 h, and was then washed three times with DI water to obtain hollow virus‐like gadolinium (HV‐Gd) nanoparticles.

### Preparation of R&HV‐Gd@ICG

HV‐Gd (100 mg) and (3‐aminopropyl) triethoxysilane (APTES, 0.75 mL, Sigma) were dissolved in anhydrous ethanol (30 mL), then were stirred for 12 h at 75 °C, and further washed three times with DI water to obtain NH_2_@HV‐Gd. Subsequently, NH_2_@HV‐Gd (50 mg) and cRGD(fK) (5 mg) were dissolved in DI water with EDC (Sigma) and NHS (Sigma) at 4 °C for 12 h. The product was washed three times with DI water. Finally, R&HV‐Gd (50 mg) and ICG (10 mg) were dissolved in DI water and stirred at 4 °C for 24 h and then washed with DI water to remove any residual ICG. Finally, R&HV‐Gd@ICG was dispersed in DI water.

### Nanoparticle Characterization

HRTEM (FEI Tecnai F20, acceleration voltage = 200 kV) and SEM (Apreo S Lovac) were used to characterize the nanoparticle morphology. Fluorescence spectra were measured using an FLS980 instrument (Edinburgh Instruments). A PerkinElmer Lambda 400 UV–vis–NIR spectrophotometer was used to measure the UV–vis spectra. The nanoparticle sizes and zeta potentials were measured using a Malvern Zetasizer (ZEN3690, Malvern, UK). The nanoparticle surface area and pore size were measured using a surface area and porosity analyzer (ASAP2050, Micromeritics Instrument Corp.). The nanoparticle elemental composition was measured using XPS (Thermo K‐alpha).

### ICG and R&HV‐Gd@ICG Photostabilities

NIR‐II fluorescence images were obtained for ICG and R&HV‐Gd@ICG by continuously laser‐irradiation at 808 nm for different times. Moreover, to evaluate the ICG and R&HV‐Gd@ICG stabilities in aqueous solutions, samples were stored in the dark at 25 °C for 96 h. At selected time points, UV–NIR absorption and fluorescence emission spectra were measured using UV–vis and fluorescence spectrometers, respectively, for the solutions.

### Nanoparticle Degradation

R&HV‐Gd@ICG was dissolved in aqueous solutions at pH 5.0, 6.5, and 7.4 for various times. At various time points, the solutions were observed using HRTEM. To further elucidate the composition of the nanoparticles degraded in vitro, R&HV‐Gd@ICG was dissolved in an aqueous solution at pH 5.0 for 24 h and was then divided into two equal parts: one of which was directly used to quantify [Gd^3+^] with ICP, and the other was initially nitrified and then used to detect [Gd^3+^]. A Malvern Zetasizer was used to further investigate the nanoparticle size in the degraded solution. The degraded samples were also observed using HRTEM. In addition, the ICG release from R&HV‐Gd@ICG was studied under different pH conditions (5.0, 6.5, and 7.4). Briefly, the R&HV‐Gd@ICG was dispersed in deionized water (1 mL). Then the solution was transferred into a dialysis bag (MWCO: 3500 Da) and dialyzed against deionized water followed by continuous stirring at the speed of 100 rpm. After 24 h, the amount of ICG in the dialysate was analyzed with a UV–vis–NIR spectrophotometer. Finally, whether degradation affected the ICG fluorescence spectral characteristics was evaluated by dissolving R&HV‐Gd@ICG in aqueous solutions at pH 5.0 and 7.4 for 24 h and then measured the absorption and NIR‐II fluorescence emission spectra of the solutions.

### Cellular Uptake

The mouse breast cancer 4T1 cell line was purchased from the American Type Culture Collection (Rockville, USA). First, 4T1 cells were placed in a 12‐well slide chamber at 1 × 10^5^ cells per well and were further cultured for 12 h. The cultured cells were treated with R&HV‐Gd@ICG, HV‐Gd@ICG, or ICG at equivalent ICG concentrations (5 µg mL^−1^) and were then reincubated for different times, digested, and measured using a flow cytometer (CytoFlexS). In addition, 4T1 cells were treated with R&HV‐Gd@ICG for 4 h, stained with 4ʹ,6‐diamidino‐2‐phenylindole (DAPI), and observed using fluorescence microscopy (Leica DM2700 P, USA). To further investigate whether the virus‐like morphology could promote cellular uptake, a smooth, hollow nanoparticle was synthesized as the control. Briefly, solid silica was obtained using the common Stöber method.^[^ ^35^ ^]^ Then, mesoporous silica was biphasally coated on the solid silica surface. The solid silica (100 mg) was redispersed in DI water (60 mL) at 60 °C with gently stirring. Then 0.72 mL of 25% triethanolamine was added to the solution, and 20 mL of a mixture consisting of cyclohexane (16 mL) and TEOS (4 mL) was laterally dropped onto the water layer and was allowed to react for 48 h. The product was treated with 0.1 m NaOH at 60 °C for 30 min and was then centrifuged at 10 000 rpm for 10 min to obtain hollow smooth mesoporous silica nanoparticles into which ICG was loaded. The 4T1 cells were treated with HV‐Gd@ICG and H‐MSN@ICG at equivalent ICG concentrations (6 µg mL^−1^). The cells were incubated for different times, stained with DAPI, and observed using fluorescence microscopy.

### Evaluation of Intracellular ROS Generation and Cell Apoptosis

4T1 cells were seeded in a 12‐well slide chamber at 1 × 10^5^ cells per well and were further cultured for 12 h and then incubated with/without R&HV‐Gd@ICG ([Gd^3+^] = 25 × 10^−6^ m) for 4 h. Then, the 12‐well plates were or were not irradiated with X‐rays (8 Gy), and the 2ʹ,7ʹ‐dichlorodihydrofluorescein diacetate (H2DCFDA) concentration was analyzed using kits according to the manufacturer's instructions (Thermo Fisher Scientific). Fluorescence images were obtained using a confocal microscope (Zeiss LSM 880+Airyscan) and were analyzed using ImageJ software. Moreover, to detect intracellular hydroxyl radical generation, R&HV‐Gd@ICG + X ray‐treated 4T1 cells were rinsed and stained with a specific •OH probe (hydroxyphenyl fluorescein; HPF; Shanghai Maokang Bio., Co.; 10 × 10^−6^ m dissolved in phosphate‐buffered saline (PBS)) for 30 min, rinsed with PBS, and imaged using a confocal microscope. Additionally, HPF was used to detect •OH generated in aqueous solution. Typically, R&HV‐Gd@ICG (1 mL) aqueous solution ([Gd^3+^] = 25 × 10^−6^ m) was X‐ray irradiated (8 Gy). Then, HPF (1 × 10^−6^ m) was immediately added, and the HPF 515 nm fluorescence intensity was detected using a fluorescence microplate reader after 15 min. DI water was used as the control. To further verify the effect of the RT sensitization on cell apoptosis, the cells were treated as just described, and cell apoptosis was detected using flow cytometry with an Annexin V‐fluorescein isothiocyanate/propidium iodide staining assay (Thermo Fisher Scientific).

### Cytotoxicity

4T1 cells were seeded in 96‐well plates at 5 × 10^3^ cells per well and were further cultured for 12 h. Subsequently, various doses of R&HV‐Gd@ICG ([Gd^3+^] = 0, 6.25, 12.5, 25, and 50 × 10^−6^ m) were added, the cells were incubated for 48 h, and cell viability was determined using a cell‐counting kit (CCK)‐8 assay. Moreover, to investigate the cytotoxicity of R&HV‐Gd@ICG with or without radiation, 4T1 cells were treated with R&HV‐Gd@ICG ([Gd^3+^] = 25 × 10^−6^ m) for 4 h and then were not or were irradiated with X‐rays (8 Gy), and cell viability was determined using methods like those just described.

### Cell Clonogenic Assay

4T1 cells were seeded in 6‐well plates at 1 × 10^3^ cells per well and were cultured for 12 h and then incubated with or without R&HV‐Gd@ICG ([Gd^3+^] = 25 × 10^−6^ m) for 4 h. Subsequently, the 6‐well plates were not or were irradiated with X‐rays (0 or 8 Gy, respectively) and then washed twice with PBS. Then, the cells were further cultured in cell culture medium for 7 d, and the culture medium was refreshed every 48 h. Once microscopic cell colonies had formed, cells from different groups were stained with crystal violet.

### Animal Models

The animal experiments were approved by the Institutional Animal Care and Use Committee of Xiamen University and were conducted in accordance with relevant guidelines (Ethics Approval: No. XMULAC20180037). Female BALB/c mice (6–8 weeks old) were purchased from Charles River Laboratories (Wilmington, MA, USA). Transgenic MMTV‐PyMT mice (FVB/N‐Tg (MMTV‐PyVT) 634Mul/J) were purchased from the Jackson Laboratory (Bar Harbor, ME, USA), and breeding was maintained using FVB/n and PyMT (MMTV‐PyMT) backgrounds. Mouse genotypes were identified at age 4 weeks. Female mice exhibiting the positive target gene were selected for NIR‐II fluorescence image‐guided tumor surgery. Subcutaneous mouse model: BALB/c mice were subcutaneously injected with 1 × 10^7^ 4T1‐Luc cells (diluted in 100 µL of PBS) in the right side of the lower limbs. Tumor volume was calculated using the standard formula: length × width^2^ × 0.52. Multiple microtumor‐bearing mice model: BALB/c mice were randomly subcutaneously injected with 5 × 10^6^ 4T1‐Luc cells (diluted in 20 µL of PBS) in the back.

### In Vitro and In Vivo MRI

The R&HV‐Gd@ICG and gadoteric acid meglumine (GAM) in vitro MRI capabilities were investigated using a 1.5‐T MRI magnet (Hantong Science and Education Equipment Co., Ltd., China). To evaluate the R&HV‐Gd@ICG and GAM MRI performances in vivo, mice previously subcutaneously injected with 4T1 cells (150–200 mm^3^) were intravenously injected with R&HV‐Gd@ICG or GAM at equivalent Gd^3+^ concentrations ([Gd^3+^] = 25 × 10^−6^ m kg^−1^). MRIs were then conducted using a 9.4‐T small animal MRI scanner (GE Healthcare, USA), and T_1_‐weighted images were obtained using an MRI scanner at different time points. T1‐weighted image parameters were as follows: TR = 1000 ms, TE = 8.5 ms, FOV = 4 × 4, matrix = 256 × 256, SI = 1.0 mm 1.0 mm^−1^, averages = 3, slices = 15, NEX = 4.

### NIR‐II Fluorescence Imaging and Biodistribution

Mice bearing subcutaneous 4T1‐Luc tumors (≈200–300 mm^3^) were randomly divided into three groups (*n* = 4) and were intravenously injected with doses of R&HV‐Gd@ICG, HV‐Gd@ICG, or ICG at the equivalent ICG dose (1.0 mg kg^−1^), respectively. The mice were then anesthetized using isoflurane (RWD Life Science), and the fluorescence signal was assessed using the NIR‐II imaging system (Series III 900/1700, Suzhou Yingrui Optical Technology Co., Ltd.) at different time points. The HV‐Gd@ICG ex vivo biodistribution was also observed. The 4T1‐Luc tumor‐bearing mice were intravenously injected with HV‐Gd@ICG and were then humanely euthanized at 48 h to collect and visualize the tumors and major organs (i.e., liver, kidney, heart, lung, spleen, skin, muscle, fat, and brain) using NIR‐II imaging.

### R&HV‐Gd@ICG Pharmacokinetics

To absolutely quantify [R&HV‐Gd@ICG], female BALB/c mice were intravenously injected with R&HV‐Gd@ICG. Then, the mice (*n* = 3) were humanely sacrificed 1, 7, 14, and 28 d postinjection, and the main organs (i.e., liver, kidney, heart, lung, and spleen) were collected and nitrated to quantify [Gd^3+^] using ICP‐MS. To determine the in vivo [R&HV‐Gd@ICG] circulating in the blood, R&HV‐Gd@ICG nanoparticles were intravenously administered through the tail vein of the BALB/c mice (*n* = 3). Blood samples (100 µL) were then collected from the posterior orbital vein of the mice at different time points postinjection and were nitrated to quantify [Gd^3+^] using ICP‐MS. To further elucidate how the nanoparticles were bodily metabolized, R&HV‐Gd@ICG was injected into the mouse tails, and mouse urine was collected at 24–36 h postinjection and was divided into two equal parts: one of which was directly used to quantify [Gd^3+^] and the other was first nitrified and then used to detect [Gd^3+^] with ICP‐MS. Finally, to assess nanoprobe degradation in vivo, 4T1 tumor‐bearing mice were not or were intravenously injected with R&HV‐Gd@ICG and were humanely sacrificed at 12 h postinjection. Then, the liver and tumor were observed using HRTEM (HITACHI H‐7650).

### Fluorescence Image‐Guided Surgery in Multiple‐Microtumor Mouse Model

Mice bearing multiple 30–60 mm^3^ 4T1‐Luc microtumors were subjected to bioluminescence imaging, and the number of microtumors was calculated. Subsequently, the mice were intravenously injected with R&HV‐Gd@ICG (ICG dose = 1.0 mg kg^−1^). Then, NIR‐II fluorescence imaging was performed 48 h postinjection, and the number of microtumors was calculated using fluorescence imaging. The consistency of the bioluminescence and fluorescence imagings in detecting the number of tumors and diagnosing pathology is the gold standard.

### Fluorescence Image‐Guided Surgery in Residual‐Tumor Mouse Model

Mice bearing 300–400 mm^3^ 4T1‐Luc tumors were randomly divided into three groups (*n* = 8), and two groups were intravenously injected with R&HV‐Gd@ICG, while the other group was intravenously injected with ICG at the equivalent ICG dose (1.0 mg kg^−1^). Tumors were resected in the R&HV‐Gd@ICG and ICG treated groups 48 and 24 h postinjection, respectively. The detailed steps are as follows. To establish a positive‐margin model, 98% of the tumor mass was resected under white light. Half of the mice in the R&HV‐Gd@ICG group did not have the residual tumor removed, and the other residual‐tumor‐bearing mice were then placed under NIR‐II imaging to observe whether residual fluorescence appeared in the surgical area. If residual fluorescence was present, the tumor was further excised until no residual signal remained. Residually fluorescent tissues were then collected for ex vivo fluorescence imaging and histological analysis. The mice were monitored every other day for body weight changes and tumor recurrence. Bioluminescence was examined 15 d postinjection. Mice exhibiting a tumor recurrence volume >1500 mm^3^ or a body weight loss of 25% were humanely euthanized.

### Fluorescence Imaging in Spontaneous Breast Cancer Transgenic Mice

MMTV‐PyVT mice exhibiting spontaneous breast cancer and wild‐type mice of the same age (6–8 weeks, *n* = 4) were intravenously injected with R&HV‐Gd@ICG (ICG dose = 1.0 mg kg^−1^). At 48 h postinjection, the mice were humanely euthanized, their skin tissues were removed to expose all the mammary tissue, and NIR‐II fluorescence imaging was performed. Subsequently, the 1st–5th breast tissue layers of both groups were resected, fluorescence imaging was performed using NIR‐II imaging, and ex vivo mean fluorescence intensity (MFI) was calculated for the tumor specimens collected from both groups. Finally, all the tissue pathologies were diagnosed by a pathologist specializing in breast cancer, and a receiver operator characteristic curve was fitted using the MFI data and the fresh tissue pathologies. In addition, 10 µm thick paraffin block sections were imaged using fluorescence flatbed scanning (Odyssey CLx). Then, adjacent 4 µm sections were prepared for H&E and IHC (*α*_v_) staining to evaluate the correlation between the fluorescence intensity and the histology in each section.

### RT Sensitization in 4T1‐Bearing Mice

50–100 mm^3^ 4T1‐tumor‐bearing mice were randomly divided into four groups (*n* = 4), which were intravenously injected with PBS and R&HV‐Gd@ICG ([Gd^3+^] = 40 × 10^−6^ m kg^−1^) and subsequently were not or were irradiated with X‐rays on days 0 and 6. The irradiated mice received X‐ray RT (6 Gy × 2) 12 h postinjection and were monitored every other day for changes in body weight and tumor volume.

### Evaluation of ROSs Generated In Vivo and Immunochemistry Analysis

50–100 mm^3^ 4T1 tumor‐bearing mice were randomly divided into four groups (*n* = 6) and were intravenously injected with PBS or R&HV‐Gd@ICG ([Gd^3+^] = 40 × 10^−6^ m kg^−1^), and X‐ray RT (6 Gy) was conducted 12 h postinjection. Half of the mice (*n* = 3) were humanely sacrificed 24 h post‐RT and the remining mice (*n* = 3) were humanely sacrificed 48 h post‐RT. Tumor tissues were collected, embedded in paraffin, cut into 4 µm thick slices, and IHC stained with [Ki67 (Abcam, ab15580 diluted 1:100), TUNEL (terminal deoxynucleotidyl transferase dUTP nick end labeling), H&E, and Caspase3 (Abcam, ab179475 diluted 1:500)], and the tissue slice ROS concentrations were analyzed using kits according to the manufacturer's instructions (Thermo Fisher Scientific). Immunofluorescence and immunochemistry images were obtained using a Leica DM2700 P (USA) and were analyzed using ImageJ software.

### Evaluation of R&HV‐Gd@ICG Biosafety

Female BALB/c mice (aged 6–8 weeks) were intravenously injected with R&HV‐Gd@ICG ([Gd^3+^] = 40 × 10^−6^ m kg^−1^), while the mice in the control group were intravenously injected with PBS. The mice were euthanized humanely euthanized 1 d, 3 d, 1 week, and 4 weeks postinjection (*n* = 4), blood was collected for hemocytological testing and measuring biochemical indexes, and major organs (heart, liver, spleen, lung, kidney, brain) were collected for histological analysis. To further determine whether R&HV‐Gd@ICG stimulated a strong immune response, additional BALB/c mice (*n* = 3) were intravenously injected with R&HV‐Gd@ICG ([Gd^3+^] = 40 × 10^−6^ m kg^−1^). The mice were humanely euthanized at 3 d and 1 week postinjection, and blood serum was collected to detect IL‐6 and TNF‐*α* concentrations using mouse ELISA kits (Thermo Fisher Scientific). The mice intravenously injected with PBS were used as the control.

### Statistical Analysis

Data were obtained from at least three independent measurements (*n* ≥ 3). All the data are presented as mean ± standard deviation (SD) unless otherwise indicated. Means were compared using a two‐sided Student's *t*‐test or one‐way analysis of variance (ANOVA), and survival was assessed using Kaplan–Meier analysis. All the experiments were performed in triplicate to ensure experimental reproducibility. *p < *0.05, *p < *0.01, *p < *0.001, and *p < *0.0001 were considered to be statistically significant with noting by *, **, ***, and ****, respectively. Graphing and linear regression were performed using Graph Pad Prism 7.0 software (Version 7.02, IBM Corp.).

## Conflict of Interest

The authors declare no conflict of interest.

## Supporting information

Supporting Information

## Data Availability

The data that support the findings of this study are available from the corresponding author upon reasonable request.
