# Supplementary material for: Biodegradable Nanoprobe for NIR‐II Fluorescence Image‐Guided Surgery and Enhanced Breast Cancer Radiotherapy Efficacy
Source: Adv Sci (Weinh). 2022 Feb 16;9(12):2104728. doi: 10.1002/advs.202104728 (PMC9036023; doi:10.1002/advs.202104728)
Supplement: Supplementary file 1 — Supporting Information [file ADVS-9-2104728-s001.pdf]

ADVANCED SCIENCE
Open Access

Supporting Information

for *Adv. Sci.*, DOI 10.1002/advs.202104728

Biodegradable Nanoprobe for NIR-II Fluorescence Image-Guided Surgery and Enhanced Breast Cancer Radiotherapy Efficacy

Rui-Qin Yang, Pei-Yuan Wang, Kang-Liang Lou, Yong-Ying Dang, Hai-Na Tian, Yang Li, Yi-Yang Gao, Wen-He Huang, Yong-Qu Zhang, Xiao-Long Liu and Guo-Jun Zhang**

ADVANCED SCIENCE
Open Access

Supporting Information

for *Adv. Sci.,* DOI: 10.1002/advs.202104728

Biodegradable Nanoprobe for NIR-II Fluorescence Image-Guided Surgery and Enhanced Breast Cancer Radiotherapy Efficacy

Rui-Qin Yang, Pei-Yuan Wang, Kang-Liang Lou, Yong-Ying Dang, Hai-Na Tian, Yang Li, Yi-Yang Gao, Wen-He Huang, Yong-Qu Zhang, Xiao-Long Liu, Guo-Jun Zhang**

Supporting Information

Biodegradable Nanoprobe for NIR-II Fluorescence Image-Guided Surgery and Enhanced Breast Cancer Radiotherapy Efficacy

Rui-Qin Yang[1,2,6 ‖], Pei-Yuan Wang[3,4 ‖], Kang-Liang Lou [1,2,6], Yong-Ying Dang[1,2,6], Hai-Na Tian[7], Yang Li[3,4], Yi-Yang Gao[1,2,6], Wen-He Huang[1,2,6], Yong-Qu Zhang[1,2,6], Xiao-Long Liu[3,4*], Guo-Jun Zhang[1,2,5,6 *]

1. Cancer Center & Department of Breast and Thyroid Surgery, Xiang'an Hospital of Xiamen University, School of Medicine, Xiamen University, Xiamen, Fujian, 361100, China;
2. Key Laboratory for Endocrine-Related Cancer Precision Medicine of Xiamen, Xiang'an Hospital of Xiamen University, Xiamen, Fujian, 361100, China;
3. Key Laboratory of Design and Assembly of Functional Nanostructures, Fujian Institute of Research on the Structure of Matter, Chinese Academy of Sciences, Fuzhou, Fujian, 350000, China;
4. The United Innovation of Mengchao Hepatobiliary Technology Key Laboratory of Fujian Province, Mengchao Hepatobiliary Hospital of Fujian Medical University, Fuzhou, Fujian, 350025, China;
5. Cancer Research Center, School of Medicine, Xiamen University, Xiamen, Fujian, 361100, China;
6. Xiamen Research Center of Clinical Medicine in Breast & Thyroid Cancers
7. Department of Biomaterials, College of Materials, Research Center of Biomedical Engineering of Xiamen & Key Laboratory of Biomedical Engineering of Fujian Province

& Fujian Provincial Key Laboratory for Soft Functional Materials Research, Xiamen University, Xiamen, Fujian, 361005, China.

***Corresponding author:** Guo-Jun Zhang, MD, PhD, Cancer Research Center and the Department of Breast-Thyroid-Surgery, Xiang'an Hospital of Xiamen University, Xiamen, Fujian, China; Email: gjzhang@xah.xmu.edu.cn, Tel: 0086-592-2889988. Xiao-Long Liu, PhD, Key Laboratory of Design and Assembly of Functional Nanostructures, Fujian Institute of Research on the Structure of Matter, Chinese Academy of Sciences, Fuzhou, Fujian, China; Email: liuxl@fjirsm.ac.cn, Tel: 0086-592-3594010.

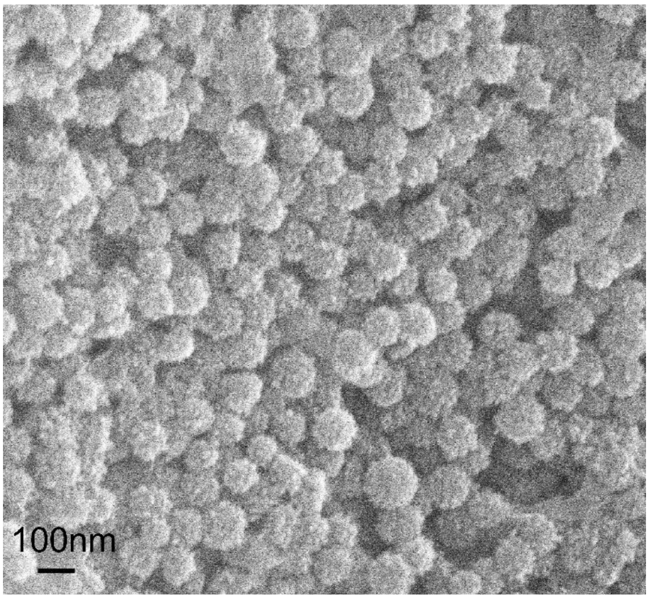

Supplemental Figure S1. The SEM image of R&HV-Gd@ICG.

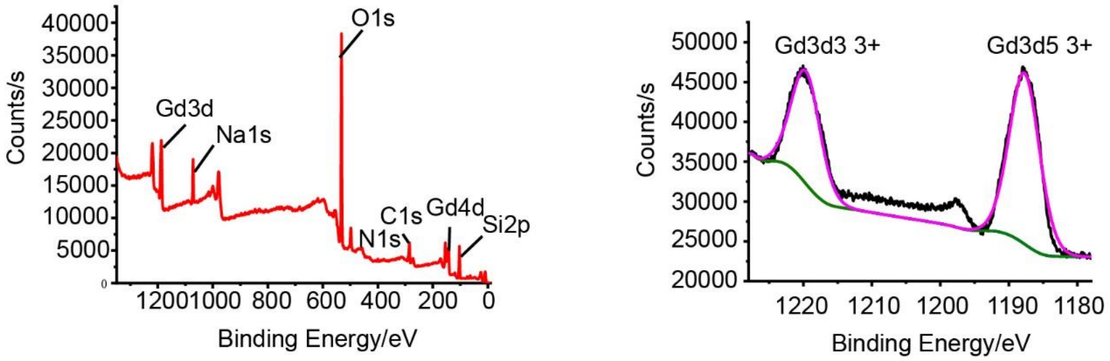

Supplemental Figure S2. The element analysis of HV-Gd nanoparticles by High-resolution X-ray photoelectron spectroscopy (left) and the Gd 3d*3/3*, Gd 3d*5/3* XPS patterns (right).

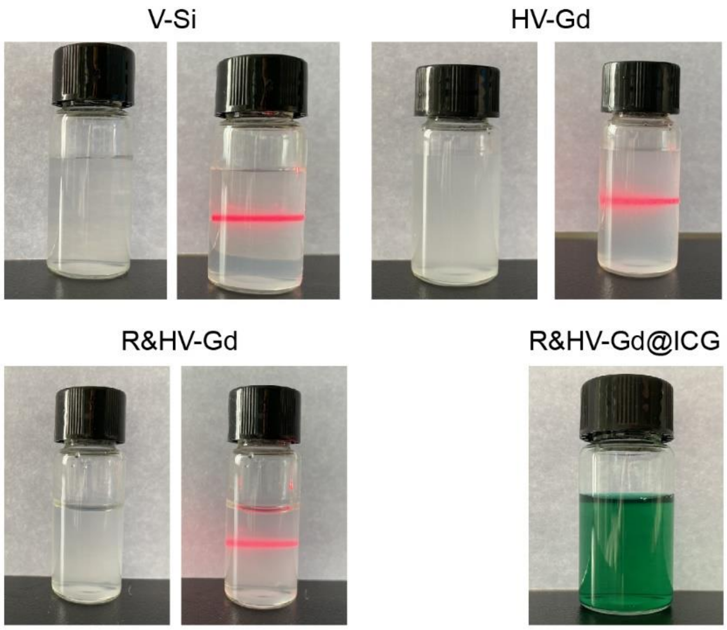

Supplemental Figure S3. Photographs and the tyndall effect of V-Si, HV-Gd, R&HV-Gd and R&HV-Gd@ICG, respectively.

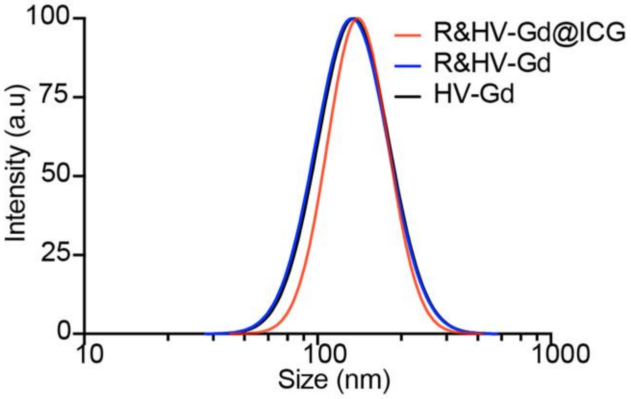

Supplemental Figure S4. The hydrate particle size of HV-Gd, R&HV-Gd and R&HV-Gd@ICG.

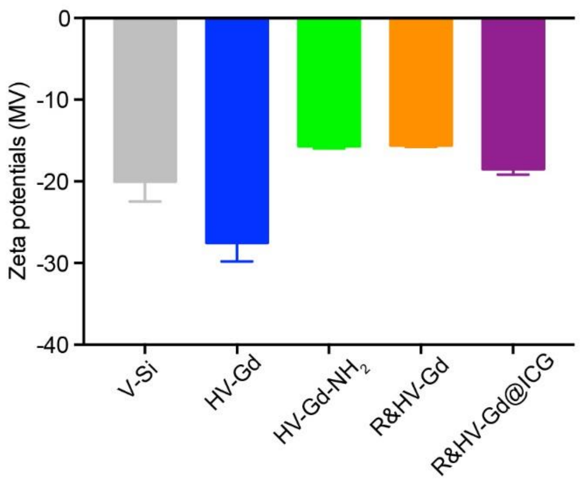

Supplemental Figure S5. Zeta potential data of V-Si, HV-Gd, HV-Gd-NH_2, R&HV-Gd and R&HV-Gd@ICG (n = 3, data were shown as means ± SD).

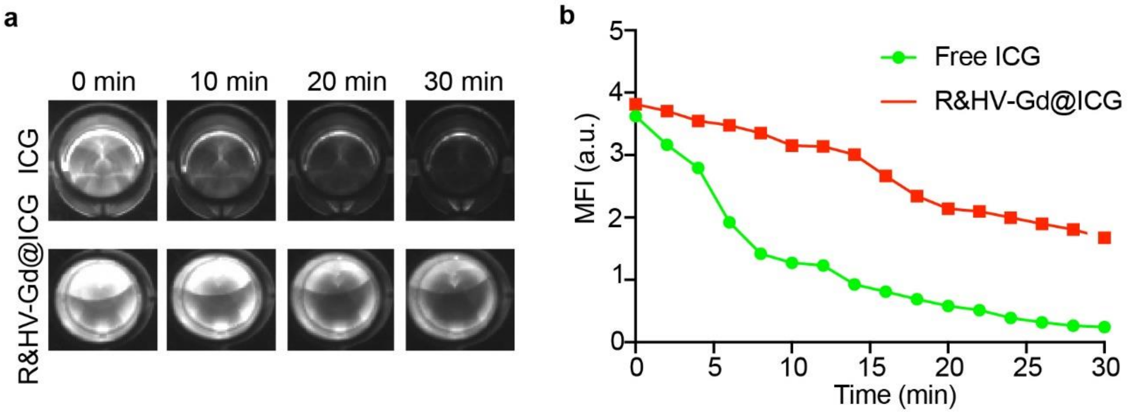

Supplemental Figure S6. Fluorescence images of ICG and R&HV-Gd@ICG after continuous laser irradiation for various periods (a), and quantitative analysis of the corresponding mean fluorescence intensity in panel a (b) (n = 3, data were shown as means ± SD).

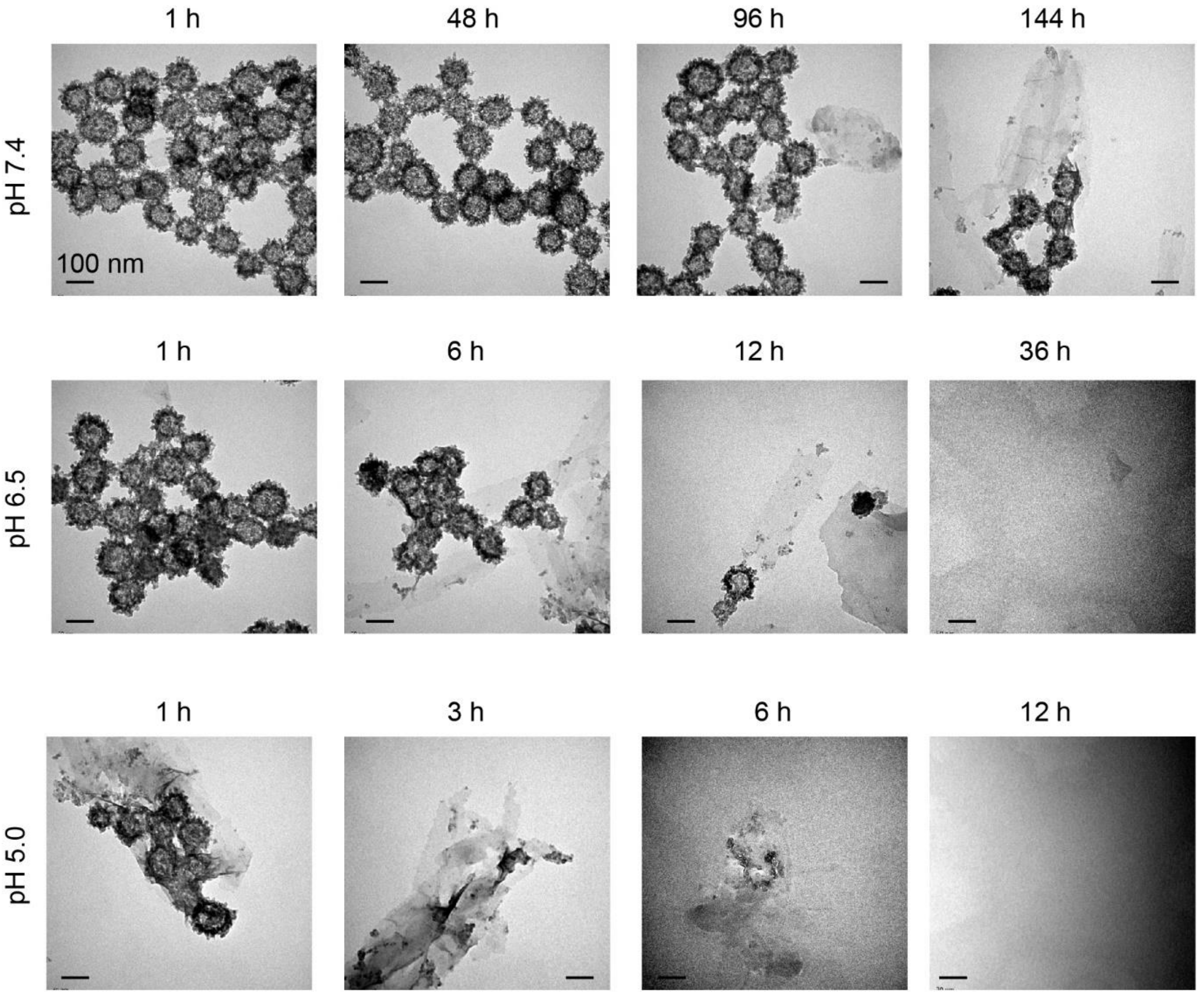

Supplemental Figure S7. TEM images of R&HV-Gd@ICG after incubation in buffers with different pH values (7.4, 6.5, and 5.0) for various periods.

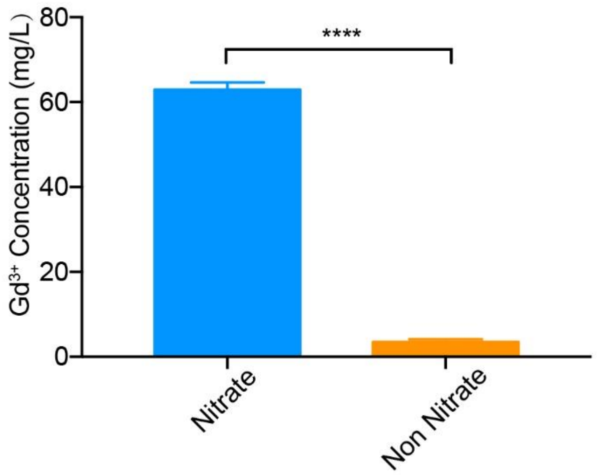

Supplemental Figure S8. The concentration of Gd^{3+} in degraded R&HV-Gd@ICG solution with/without nitrification (n = 3, data were shown as means ± SD, statistical significance is determined by two-tailed unpaired t-test, ****p<0.0001).

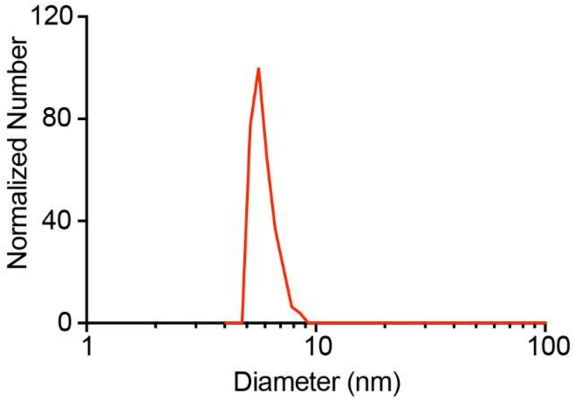

Supplemental Figure S9. The hydrate particle size of R&HV-Gd@ICG degraded products.

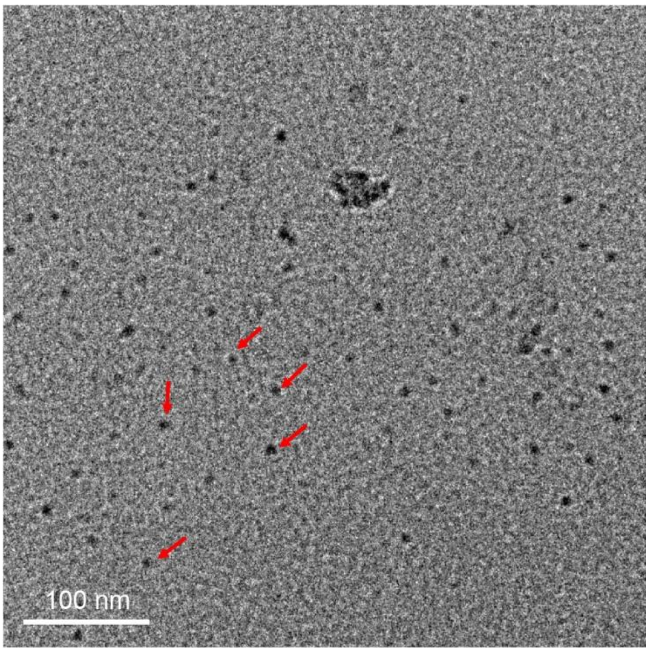

Supplemental Figure S10. The HRTEM image of R&HV-Gd@ICG degraded products and the red arrows represent degraded nanoparticles.

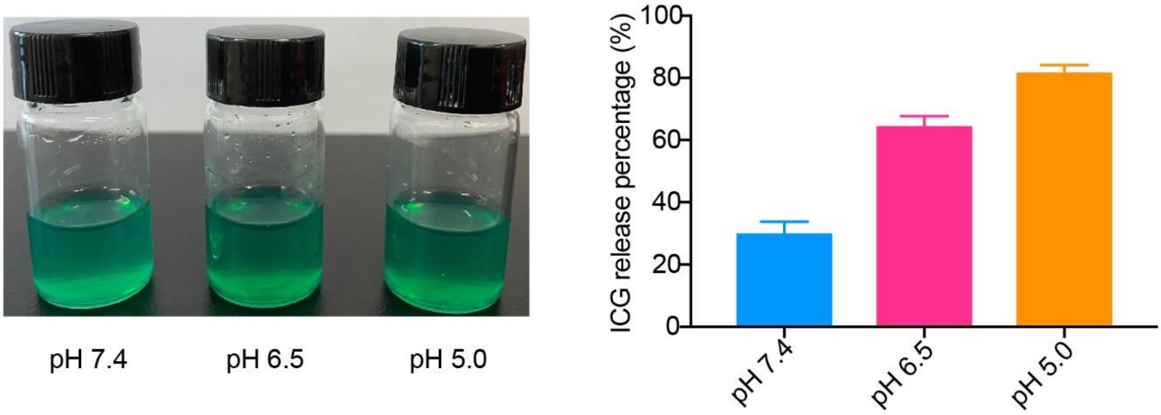

Supplemental Figure S11. The photographs of R&HV-Gd@ICG after 24 h incubation at different pH solutions(left), and the amount of ICG released from R&HV-Gd@ICG after 24 h incubation at different pH solutions (right) (n = 3, data were shown as means ± SD).

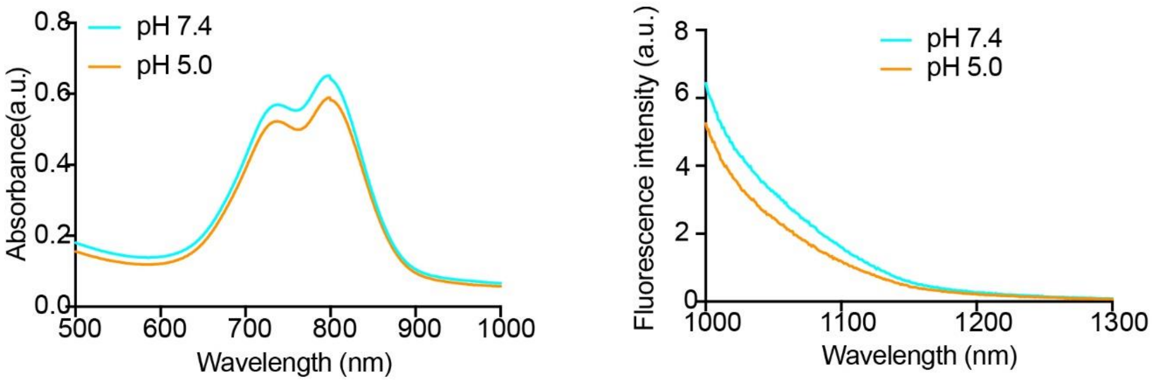

Supplemental Figure S12. The UV–vis-NIR absorption spectra (left) and NIR-II fluorescence emission spectra (right) of R&HV-Gd@ICG, which were incubated in pH 5.5 and 7.4 solutions for 24 hours, respectively.

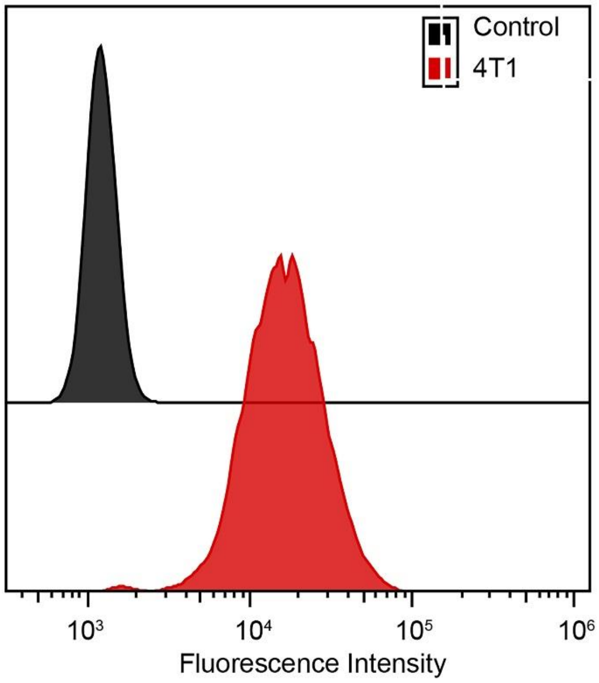

Supplemental Figure S13. Flow cytometry analysis of the $\alpha_v\beta_3$ expression in 4T1 cell lines.

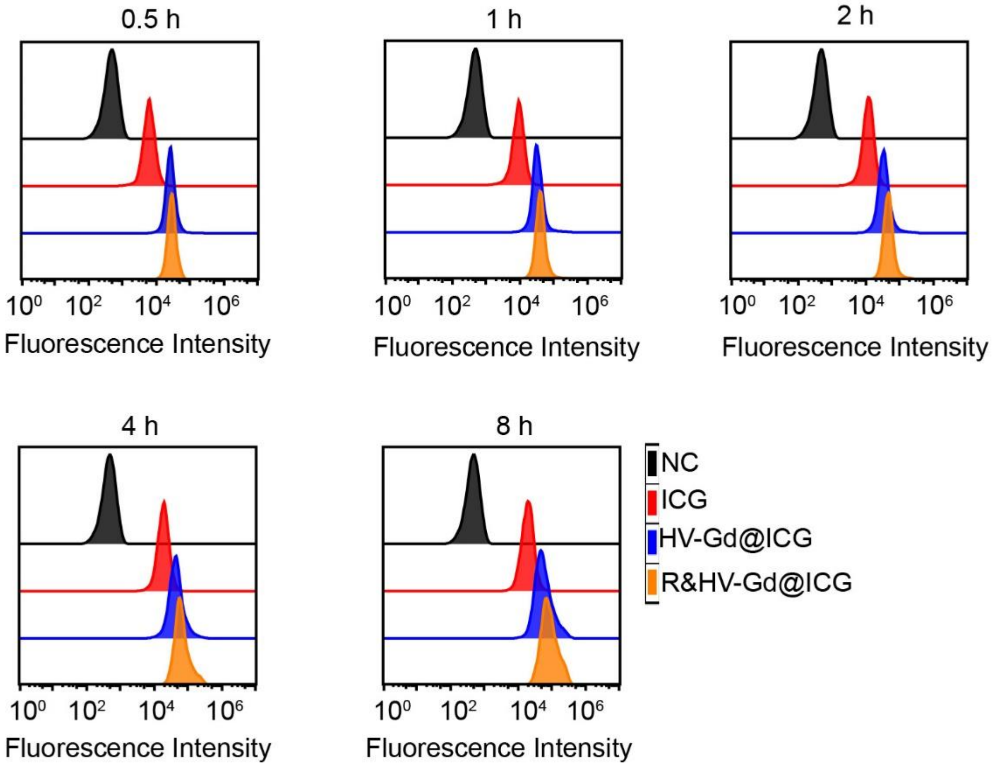

Supplemental Figure S14. Flow cytometry analysis of 4T1 cells incubated with PBS, ICG, R&HV-Gd, and R&HV-Gd@ICG for various periods of time.

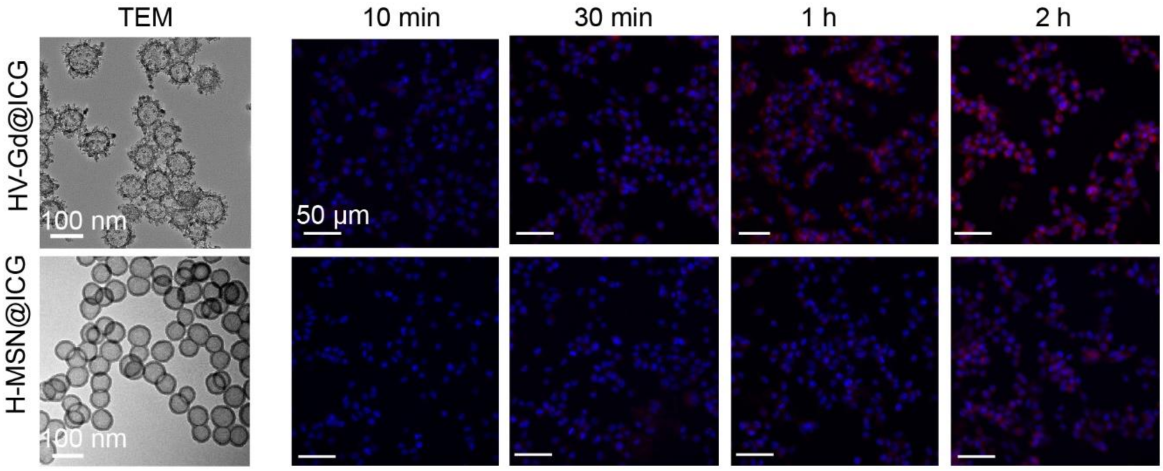

Supplemental Figure S15. The TEM images of HV-Gd@ICG and H-MSN@ICG (left); the fluorescence microscope images of 4T1 cells incubated with HV-Gd@ICG and H-MSN@ICG for various periods of time (right).

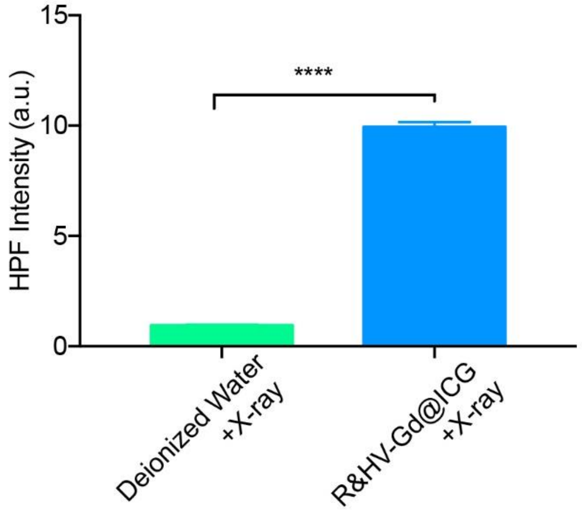

Supplemental Figure S16. The fluorescence intensity of HPF in the deionized water and R&HV-Gd@ICG solution with X-ray irradiation, respectively (n = 3, data were shown as means ± SD, statistical significance is determined by two-tailed unpaired t-test, ****p<0.0001).

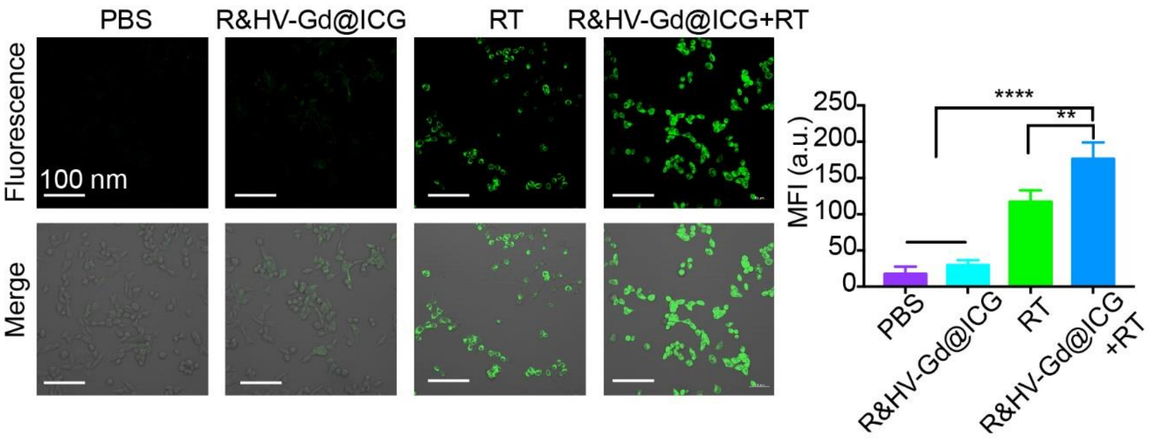

Supplemental Figure S17. Confocal laser scanning microscope images of intracellular •OH stained by HPF staining in 4T1 cells treated with PBS, R&HV-Gd@ICG with or without X-ray (8 Gy)(left), and quantitative analysis of intracellular HPF fluorescence intensity (right) (n = 3, data were shown as means ± SD, statistical significance is assessed using one-way ANOVA followed by Tukey's multiple comparisons test, **p < 0.01, ****p < 0.0001).

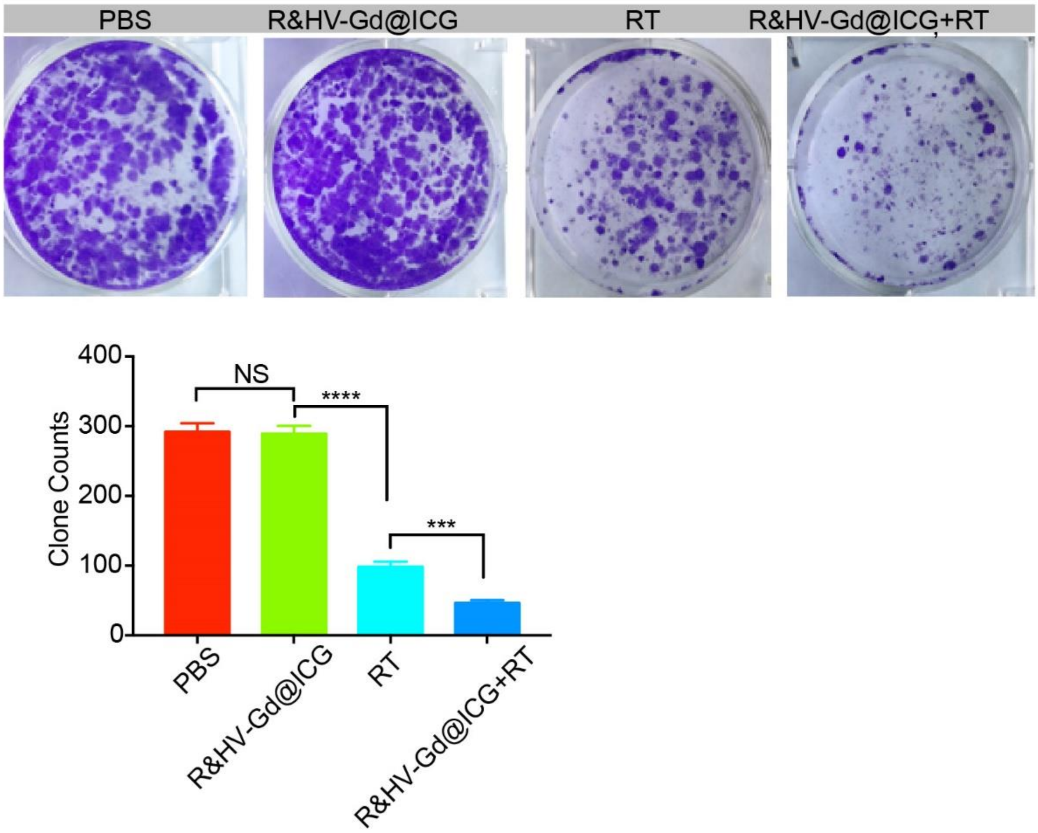

Supplemental Figure S18. Colony of 4T1 cells treated with PBS, R&HV-Gd@ICG, RT and R&HV-Gd@ICG + RT (upper), and quantification of clone counts (lower) (n = 3, data were shown as means ± SD, statistical significance is assessed using one-way ANOVA followed by Tukey's multiple comparisons test, ***p<0.001, ****p<0.0001).

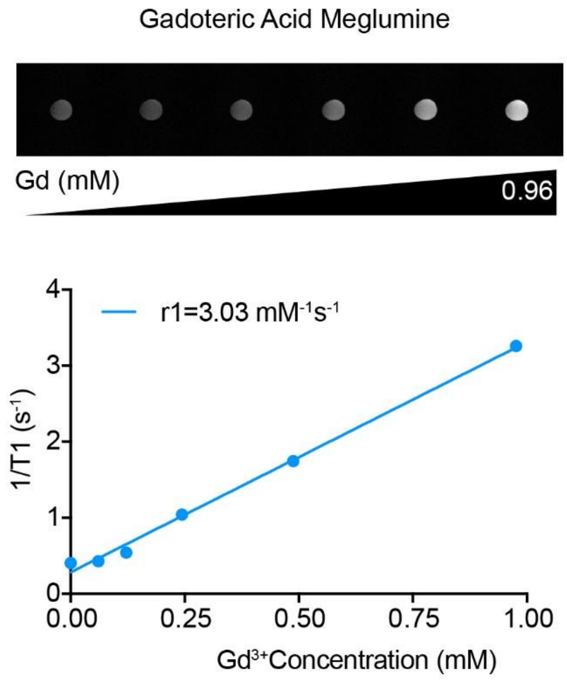

Supplemental Figure S19. T1-weighted MR images (upper) and the longitudinal relaxivities (r1) values (lower) of the Gadoteric Acid Meglumine recorded using 1.5 T MR scanner.

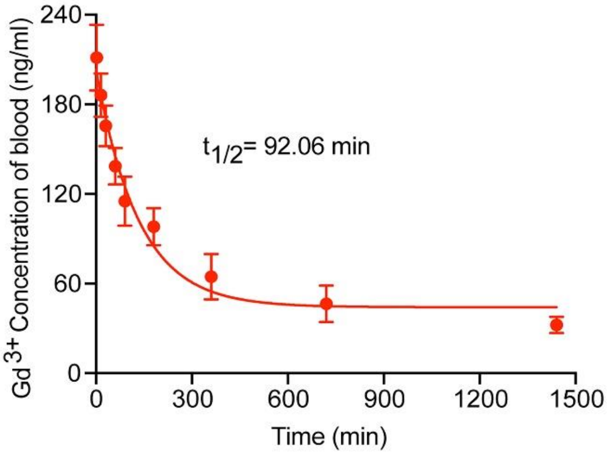

Supplemental Figure S20. Blood concentration versus time curve of R&HV-Gd@ICG nanoprobe in the mice (n = 3).

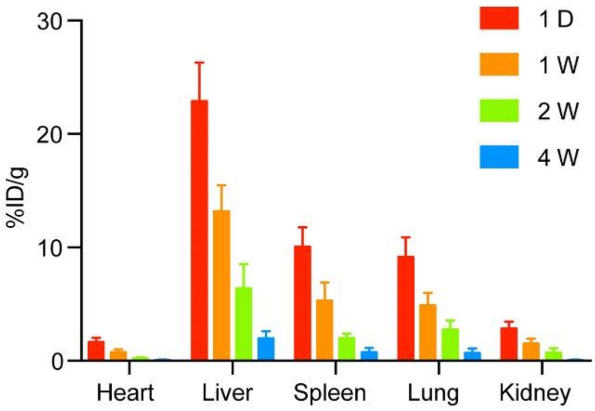

Supplemental Figure S21. Time-dependent biodistribution profile of different organs after intravenous injection R&HV-Gd@ICG nanoprobe for different periods (n = 3, data were shown as means ± SD).

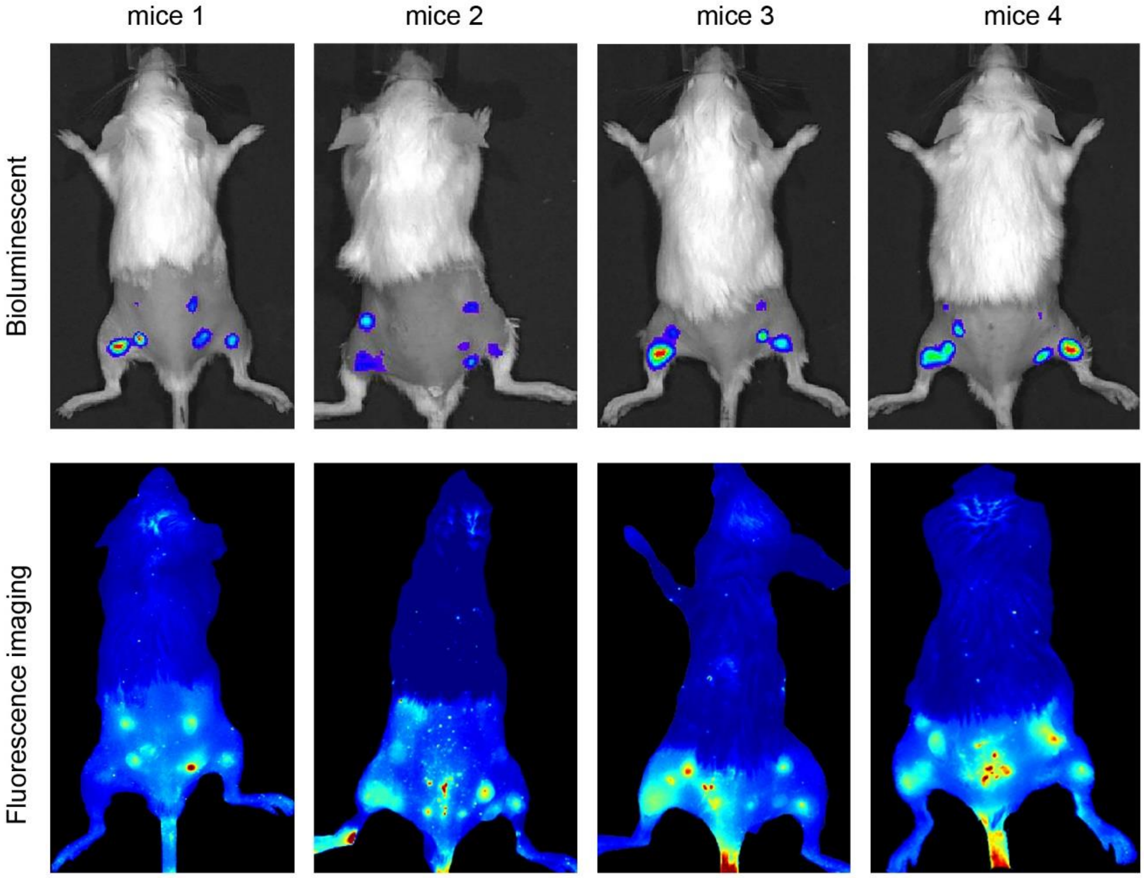

Supplemental Figure S22. Preoperative bioluminescent and fluorescent images of the

multiple micro-tumor bearing mice after 48 h injection of the R&HV-Gd@ICG nanoprobe.

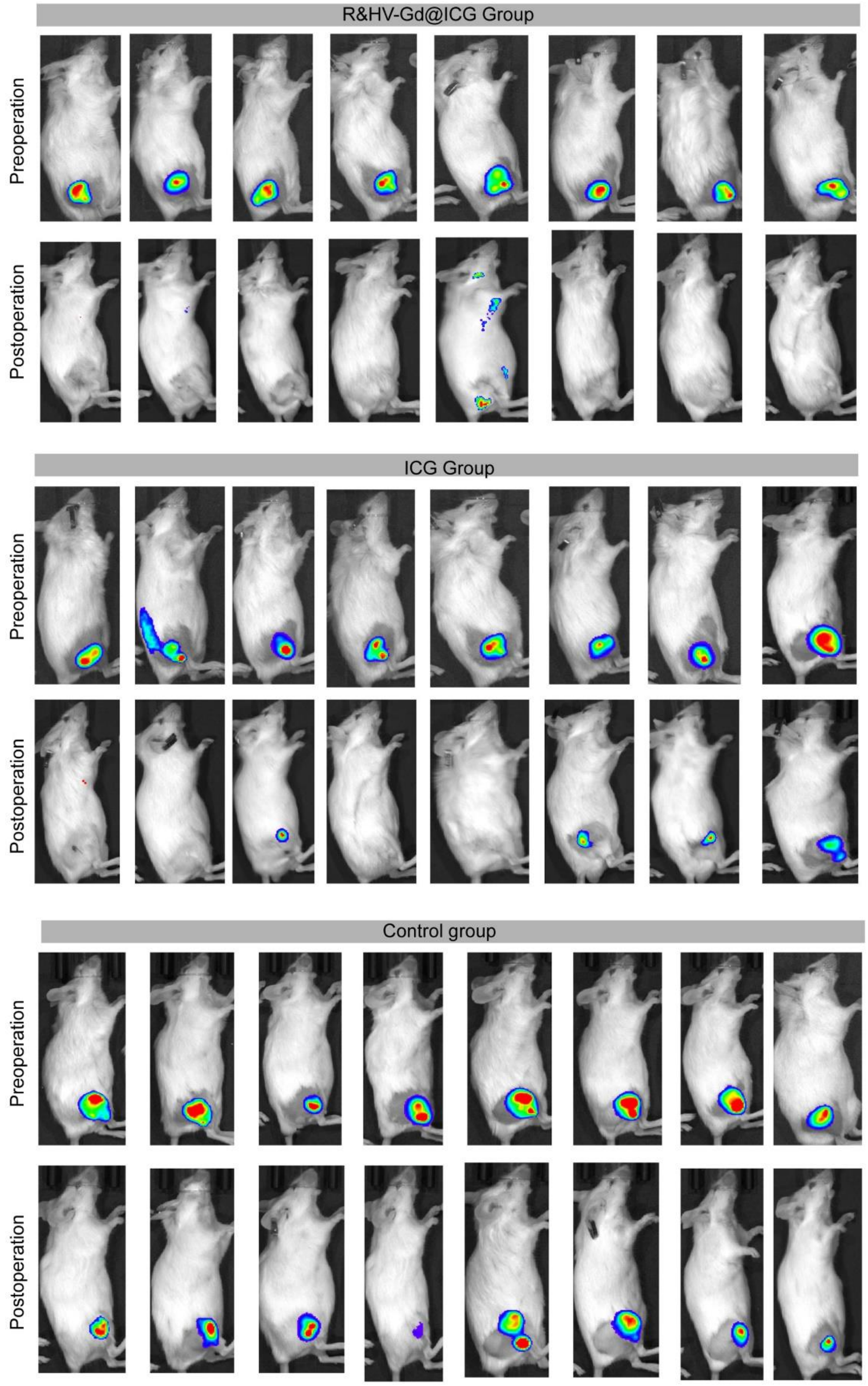

Supplemental Figure S23. Preoperative and postoperative bioluminescent images of the

4T1-tumor bearing mice under tumor resection surgery after injected R&HV-Gd@ICG and ICG.

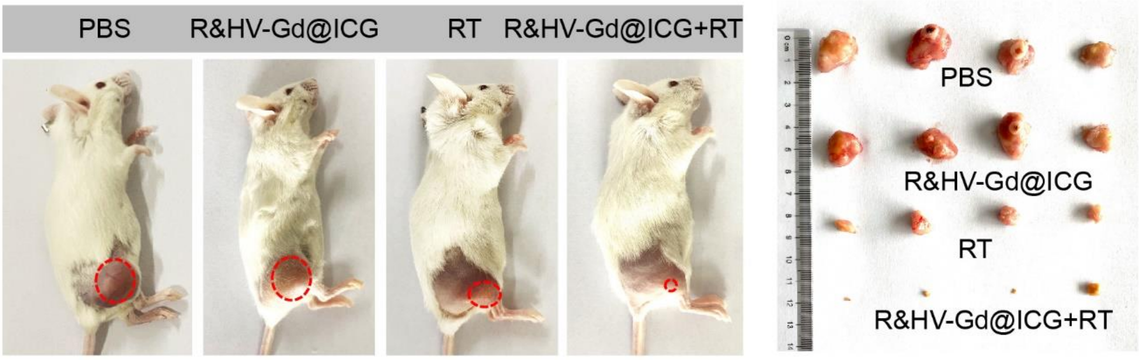

Supplemental Figure S24. Photographs of representative mouse (left) and tumors harvested from mice (right) (*n* = 4) treated with PBS, R&HV-Gd@ICG, RT and R&HV-Gd@ICG+ RT.

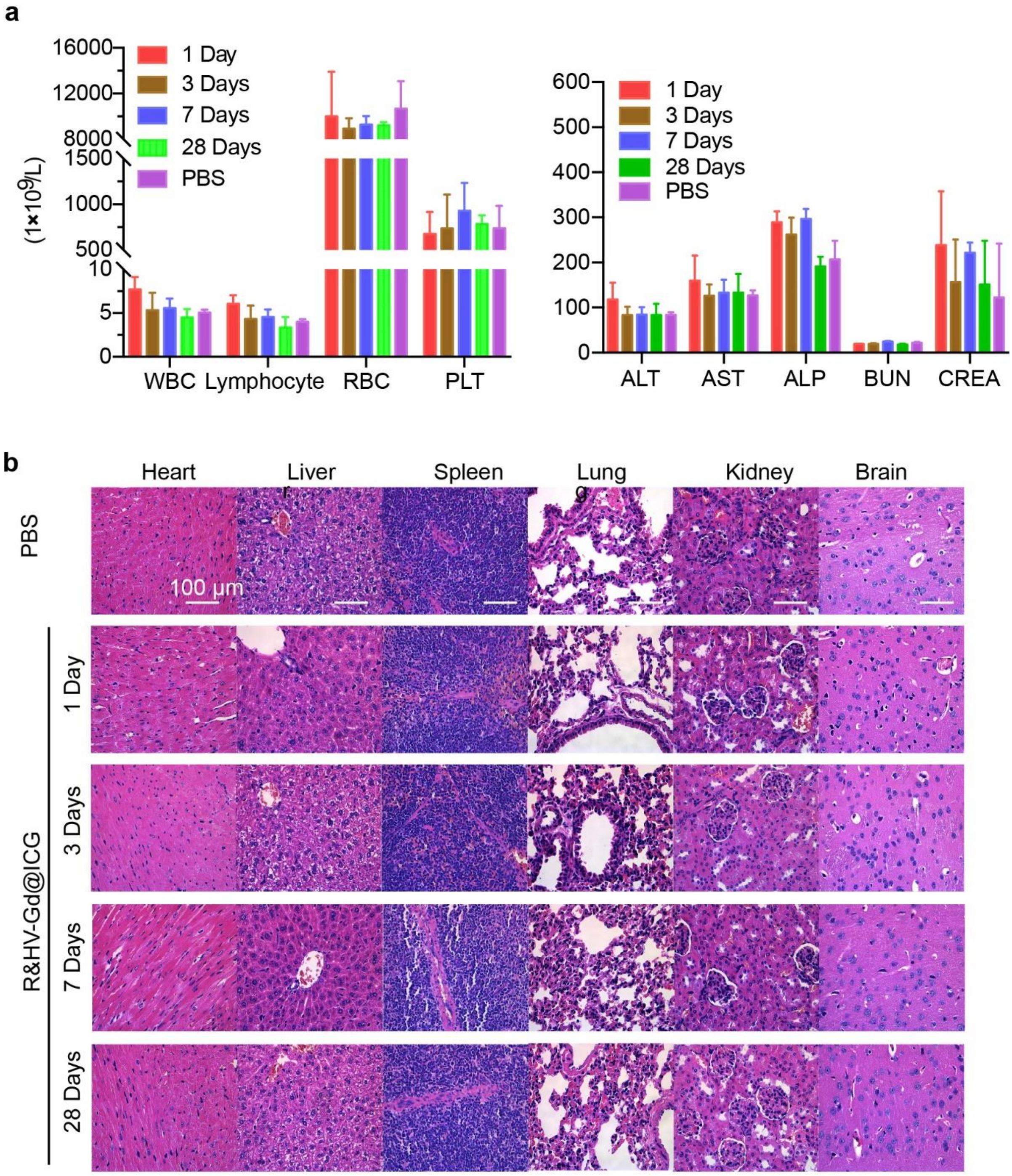

Supplemental Figure S25. Blood cytology, liver and kidney function indexes (a) H&E-stained images (b) of major organs from healthy control mice and mice after intravenous injection of R&HV-Gd@ICG nanoprobe at different time points (n = 3, data were shown as means ± SD).

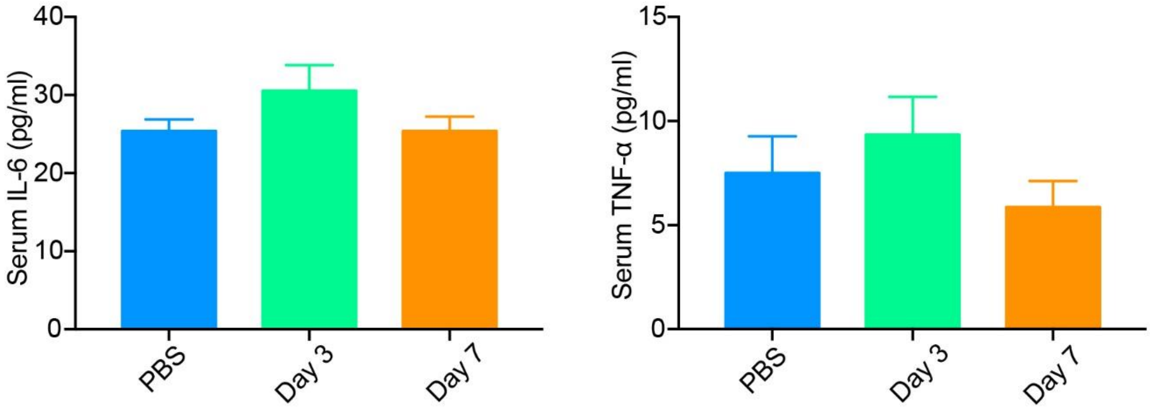

Supplemental Figure S26. The mice serum levels of IL- 6 and TNF- α after treated with R&HV-Gd@ICG for different days, PBS treatment was set as a control (*n* = 3, data were shown as means ± SD).
